# Antisense Oligonucleotidesin Genetic Epilepsies and Developmental and Epileptic Encephalopathies: Molecular Mechanisms, Translational Evidence and Disease-Modifying Potential

**DOI:** 10.3390/biology15212010

**Published:** 2026-11-05

**Authors:** Antonio Leo, Roberta Roberti, Pio Francesco Vono, Valentina Rajone, Valeria Di Stefano, Maria Pisano, Maria Cristina Caroleo, Emilio Russo

**Affiliations:** 1 Department of Health Sciences, University “Magna Graecia” of Catanzaro, 88100 Catanzaro, Italy; r.roberti@unicz.it (R.R.); maria.pisano93@gmail.com (M.P.); mariacristina.caroleo@unicz.it (M.C.C.); erusso@unicz.it (E.R.); 2 Clinical Research Unit for Innovation, Support and EthicsResearch Center, University “Magna Graecia” of Catanzaro, 88100 Catanzaro, Italy; 3 Independent Researcher, 88100 Catanzaro, Italy; vono.piofrancesco@gmail.com; 4 Unit of Clinical Pharmacology and Pharmacovigilance, AOU R. Dulbecco, Magna Graecia University, 88100 Catanzaro, Italy; valentinarajone99@gmail.com (V.R.); valedistefano01@hotmail.it (V.D.S.)

**Keywords:** antisense oligonucleotides, developmental and epileptic encephalopathy, Dravet syndrome, Angelman syndrome, *SCN1A*, *SCN2A*, *UBE3A*, *GNAO1*, intrathecal delivery, disease modification

## Abstract

In genetic developmental and epileptic encephalopathies, disability results from both the molecular defect and epileptic activity. Antiseizure medications modulate pathways involved in seizure initiation and propagation but generally do not correct the disease-causing molecular alterations that contribute to epileptic susceptibility and neurodevelopmental dysfunction. Antisense oligonucleotides (ASOs) modify the splicing of ribonucleic acid (RNA) transcripts, promote RNA degradation, or reactivate silenced alleles. This narrative review examines variant-guided mechanisms, translational evidence, clinical outcomes, and barriers to implementation. ASO programs targeting *SCN1A*, *SCN2A*, *SCN8A*, *KCNT1*, *GNAO1*, and *UBE3A* range from individualized treatments to phase 3 trials. Published uncontrolled zorevunersen studies report preliminary clinical signals in Dravet syndrome, whereas sponsor-reported results from the randomized, sham-controlled EMBRAVE Part A study suggest seizure reduction with elsunersen in *SCN2A*-related disease. Conversely, sponsor-reported phase 3 Aspire topline results indicate that apazunersen did not meet its primary cognitive or key secondary multidomain endpoint in Angelman syndrome. These findings are not equivalent in evidentiary strength. Intrathecal delivery, heterogeneous central nervous system (CNS) exposure, target-dosage sensitivity, and compound-specific toxicity influence the therapeutic window. Disease modification requires controlled longitudinal demonstration of meaningful outcomes beyond target engagement or seizure reduction, considering development, adaptive functioning, survival, and treatment burden.

## Simple Summary

Certain genetic epilepsies are caused by a change in a single gene, which leads to seizures and, in many children, developmental delay. Current antiseizure medications reduce seizures without correcting the genetic cause. Antisense oligonucleotides are short synthetic molecules that bind to the cell’s working copy of a gene and alter how its instructions are read. They can increase a deficient protein, reduce an overactive one, or activate a healthy gene copy that is normally silent. Compounds for Dravet and Angelman syndromes have reached late-stage trials, and some patients have received molecules designed for their specific variant. Treatment selection depends on whether the variant reduces or increases gene function, making genetic diagnosis essential. A central question is whether fewer seizures translate into better development. Challenges include repeated injections into the fluid surrounding the brain and spinal cord, uneven distribution across brain regions, and potential side effects. This review synthesizes current evidence and identifies what remains to be established: long-term benefits for development and daily functioning, alongside safety and treatment burden, beyond seizure reduction alone.

## 1. Rationale: From Seizure Suppression to Molecular Rescue

Genetic developmental and epileptic encephalopathies (DEEs) are characterized by seizures and developmental impairment whose relationship cannot be explained by seizure burden alone. The International League Against Epilepsy (ILAE) distinguishes the developmental consequences of the underlying etiology from the additional effects of epileptic activity, including interictal epileptiform discharges. Both processes may contribute to the same individual, with their relative importance varying across disorders and over time. This distinction has direct therapeutic implications: suppression of seizures may reduce an aggravating component of developmental dysfunction while leaving the primary biological disturbance unresolved. Conversely, persistent disability after seizure control does not negate the clinical value of controlling epilepsy. Throughout this review, disease modification denotes a treatment-related alteration in the course of the disorder, demonstrated by longitudinal clinical evidence rather than inferred from engagement with the molecular target. Developmental outcome and seizure outcome therefore require separate assessment, even when they arise from interacting mechanisms [1].

Antiseizure medications (ASMs) remain indispensable for reducing seizures and treating status epilepticus, alongside rehabilitation and management of associated disabilities. Contemporary care of Dravet syndrome, a Sodium Voltage-Gated Channel Alpha Subunit 1 (SCN1A)-related developmental and epileptic encephalopathy that begins in the first year of life, illustrates this broader approach, combining syndrome-appropriate maintenance treatment, individualized emergency plans and assessment of cognitive, behavioral, motor and sleep difficulties. Nevertheless, reducing neuronal excitability does not generally restore the expression or function of a disease-causing gene product. The unmet need extends beyond additional seizure reduction to treatments that improve the neurological course by intervening in the causal mechanism. Such treatments must be evaluated alongside established care, because molecular targeting does not remove the immediate need for effective seizure management [2].

A molecular diagnosis becomes therapeutically informative when the functional consequences of the variant are understood. Loss of functional protein, excessive protein activity, abnormal ribonucleic acid (RNA) processing, and interference by a mutant protein can require different interventions. Even within a single gene, the appropriate direction of treatment may differ between patients. Sodium Voltage-Gated Channel Alpha Subunit 2 (*SCN2A*)-related disorders illustrate the point: early infantile epilepsy has been associated with gain-of-function variants, whereas other presentations involve loss of function, so that the two groups call for opposite interventions. Age at onset is not, however, a substitute for functional assessment; Section 3 develops this decision axis in full. A strategy that lowers gene expression must therefore be justified by the relevant disease mechanism and the amount of normal function that needs to be preserved [3].

Antisense oligonucleotides (ASOs) provide a means of adjusting gene expression through sequence-directed interactions with RNA. These synthetic, single-stranded molecules bind complementary sequences in precursor messenger RNA, mature messenger RNA, or regulatory non-coding transcripts. Their effects depend on the binding site and the chemical design: some recruit ribonuclease H1 (RNase H1) to cleave the bound RNA, others redirect RNA processing by steric occupancy alone, as developed in Section 2. Thus, antisense pharmacology can support either suppression or augmentation of protein expression [4]. Blocking a non-productive splicing event can increase the proportion of transcripts that encode functional protein, whereas reducing a repressive antisense transcript can permit expression from a silenced allele. These mechanisms have been demonstrated experimentally by modulating non-productive splicing and reactivating paternal *Ube3a* expression in experimental Angelman syndrome models, respectively [5,6].

The clinical feasibility of treating a genetic neurological disorder through intrathecal splice modulation was established by nusinersen in spinal muscular atrophy. Nusinersen promotes inclusion of exon 7 in Survival of Motor Neuron 2, Centromeric (*SMN2*) transcripts, increasing production of full-length survival motor neuron protein. In the final analysis of the sham-controlled ENDEAR trial, 51% of evaluable nusinersen-treated infants achieved a motor-milestone response, compared with none in the control group; the hazard ratio for death or permanent assisted ventilation was 0.53. These findings demonstrated that an RNA-directed intervention could affect functional and survival outcomes. They also provided a clinical precedent for repeated intrathecal administration. Transfer to genetic epilepsies nevertheless requires separate validation because the affected neuronal populations, spatial distribution of pathology and relationships between gene dosage and function differ [7].

Experimental and clinical epilepsy studies now provide evidence specific to this therapeutic rationale. In a Dravet syndrome mouse model, increasing productive Scn1a expression with an ASO reduced electrographic seizures and the incidence of sudden unexpected death in epilepsy (SUDEP). This finding connected correction of a molecular deficit with clinically relevant outcomes in an experimental system [8]. Subsequent open-label studies of zorevunersen in children and adolescents with Dravet syndrome reported reductions in convulsive seizures and signals of improvement in adaptive behavior and quality of life [9]. Human experience with *SCN2A*-directed treatment has also demonstrated the feasibility of administering a transcript-lowering ASO to a patient with functionally characterized gain-of-function disease [10]. These observations support further clinical testing, while differences in study design, concomitant treatment and outcome ascertainment limit comparisons and causal interpretation.

The translation of molecular rescue into sustained benefit also depends on exposure and treatment timing. Intrathecal administration facilitates central nervous system delivery, but experimental imaging shows that distribution, cellular uptake and clearance follow distinct anatomical and temporal patterns. Administration into cerebrospinal fluid does not ensure equivalent target engagement throughout the brain [11]. Earlier intervention may also influence the extent of functional recovery: in ENDEAR, shorter disease duration was associated with greater benefit, although the corresponding therapeutic windows in genetic epilepsies must be established independently [7]. Disease-modifying potential must consequently be assessed for a specific ASO, molecular mechanism, dose and stage of disease. The relevant standard is a sustained improvement in the clinical course, supported by credible comparative evidence and weighed against adverse effects and the cumulative burden of treatment. The field has now reached the point at which this standard can be applied rather than anticipated, because the first phase 3 programs in genetic developmental and epileptic encephalopathies have begun to report. Section 2 and Section 3 set out the pharmacology and the genotype-to-mechanism logic, Section 4 grades the evidence program by program, and Section 5 and Section 6 address delivery, safety and the obstacles that remain.

### 1.1. Scope and Evidence Selection

This narrative review synthesizes experimental studies, peer-reviewed clinical reports, trial registries, and identified sponsor disclosures by molecular mechanism, target, clinical maturity, and safety. Priority was given to direct RNA-modulation evidence in genetic epilepsies and to mechanistically relevant central nervous system (CNS) delivery and outcome literature. Four complementary targeted PubMed searches were conducted on 30 October 2026, during revision, retrieving 1005 records and 884 unique PMIDs after deduplication. The searches were a retrospective verification of the evidence base first made publicly available on or before 28 September 2026; they did not extend the original evidence cutoff. Full search expressions, retrieval counts, and evidence selection principles are provided in Supplementary File S1. In addition to PubMed, targeted searches of ClinicalTrials.gov were performed using relevant gene names, ASO compound and program names, and clinical trial identifiers. Dated sponsor disclosures were identified through official company websites for programs lacking complete peer-reviewed clinical reports. Trial registrations were used to document study design and registration status, whereas sponsor communications were classified as non-peer-reviewed evidence. Only information publicly available on or before the 28 September 2026, evidence cutoff was considered eligible for inclusion. This is a narrative review: no exhaustive duplicate screening, formal risk-of-bias assessment, or meta-analysis was performed, and potential selection and publication bias remain limitations. The clinical emphasis on severe developmental and epileptic encephalopathies is distinguished from the broader mechanistic relevance to genetic epilepsies in Section 1.2.

### 1.2. Genetic Epilepsies Beyond the DEE Spectrum: Current Translational Limitations

Genetic epilepsies include phenotypes without prominent developmental impairment, such as self-limited neonatal and infantile epilepsies. The ILAE distinguishes these from developmental and epileptic encephalopathies while recognizing that variants in the same gene, including *KCNQ2* and *SCN2A*, can cause markedly different phenotypes [3,12]. Clinical ASO development discussed here has concentrated on severe DEEs and related neurodevelopmental disorders. In less severe conditions, spontaneous remission or seizure control with established therapies may alter the anticipated benefit–risk balance relative to repeated intrathecal administration, prolonged RNA modulation, and possible neurological toxicity. These are plausible clinical considerations, not established explanations of individual sponsor decisions. Extension to non-DEE phenotypes requires a functionally validated, RNA-amenable mechanism and favorable benefit–risk balance assessed against natural history and available treatments [13].

## 2. Molecular Pharmacology and Mechanisms of Action

### 2.1. Chemistry, Architecture and the Basis of Antisense Activity

ASOs are synthetic, single-stranded oligonucleotides, typically 15–30 nucleotides long, that recognize complementary RNA sequences through Watson–Crick base pairing. Their targets include precursor messenger RNA (pre-mRNA), mature messenger RNA and regulatory non-coding RNA. Sequence complementarity determines target recognition, whereas chemical architecture and binding position determine the resulting pharmacological effect. The term antisense therefore encompasses interventions that decrease gene expression, redirect splicing or increase functional protein output. These effects do not require correction of the underlying genomic variant [4].

Chemical modification is necessary to obtain useful stability, tissue exposure and activity. Phosphorothioate substitution replaces a non-bridging oxygen in the phosphate backbone with sulfur, increasing resistance to nucleases and altering interactions with proteins. These interactions influence distribution and cellular uptake but can also contribute to toxicity. Sugar modifications, including 2′-O-methoxyethyl (2′-MOE), locked nucleic acid and constrained ethyl, modify RNA affinity and metabolic stability. Phosphorodiamidate morpholino oligomers use a different, uncharged backbone and support steric-blocking mechanisms. Chemical classes are consequently not interchangeable: changes in affinity, protein binding and intracellular trafficking can alter both potency and tolerability [4].

The effects of backbone and sugar modifications should, however, be distinguished. Phosphorothioate linkages influence protein binding and tissue distribution, whereas 2′-sugar modifications alter RNA-binding affinity, nuclease resistance, and intracellular persistence. Their combined effects on cellular uptake, endosomal trafficking, pharmacological potency, and toxicity are compound-dependent [4,14].

RNase H1-dependent ASOs require formation of an RNase H1-compatible duplex of RNA and deoxyribonucleic acid (DNA), typically achieved through a gapmer architecture containing a central DNA segment flanked by chemically modified nucleotides. The DNA region supports RNase H1 recognition and cleavage of the RNA strand, while the modified flanks enhance affinity and stability. In contrast, fully 2′-modified steric-blocking ASOs can modulate RNA processing through sequence-specific occupancy without recruiting RNase H1 or inducing target-RNA cleavage [4,15]. Experimental studies demonstrate that RNase H1-dependent RNA cleavage can operate in both the nucleus and cytoplasm, allowing degradation of nuclear transcripts as well as mature messenger RNA. It should therefore not be described as an exclusively nuclear process. Target accessibility and the availability of productive intracellular drug, rather than sequence complementarity alone, determine whether efficient cleavage occurs [15].

### 2.2. Mechanistic Strategies: Lowering, Splice Redirection and Allele Unsilencing

**Figure 1:**
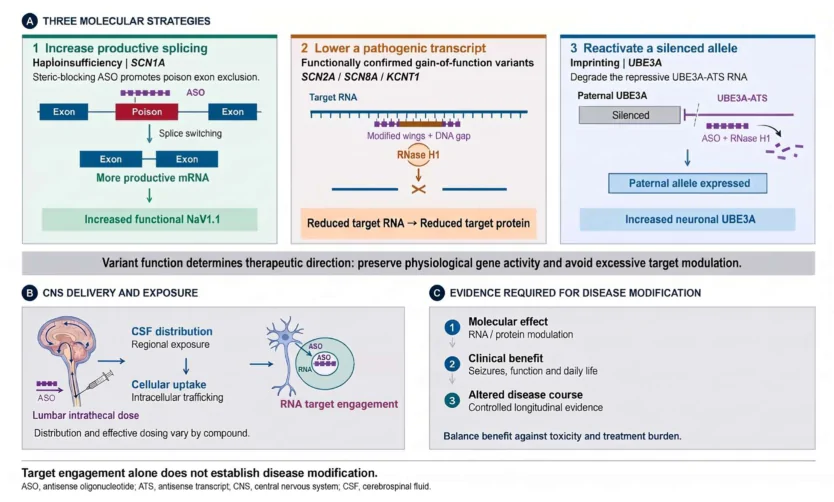
Molecular strategies, central nervous system delivery and evaluation of antisense oligonucleotides in genetic epilepsies. (**A**) Representative mechanisms matched to the underlying molecular defect. In *SCN1A* haploinsufficiency, steric-blocking splice modulation reduces inclusion of a nonproductive poison exon, thereby increasing productive transcript abundance and functional NaV1.1 expression. RNase H1-recruiting gapmer ASOs reduce target mRNA abundance and protein expression in selected gain-of-function disorders involving *SCN2A*, *SCN8A*, or *KCNT1*. By contrast, degradation of *UBE3A-ATS* relieves silencing of the paternal *UBE3A* allele and increases neuronal UBE3A protein expression. The direction and magnitude of modulation must preserve sufficient physiological function and avoid excessive target modulation. These strategies are variant- and mechanism-dependent and should not be extrapolated to all pathogenic variants within the respective genes [5,6,8,16,22,23]. (**B**) Following intrathecal administration, ASOs undergo regional CNS distribution, cellular uptake, and intracellular trafficking before productive engagement of the target RNA can occur. CSF or tissue exposure does not necessarily establish intracellular target engagement, and distribution and dosing requirements are compound-specific [11,18]. (**C**) Proposed evidentiary framework distinguishing molecular activity, clinically meaningful benefit, and alteration of disease trajectory. Target engagement alone does not establish disease modification, which requires controlled longitudinal evidence of a favorable change in clinically relevant outcomes or disease course. This panel represents a conceptual synthesis rather than a validated surrogate-endpoint framework. The figure does not imply regulatory approval or equivalent efficacy, safety, or clinical maturity across programs. Anatomical structures, exon lengths, and oligonucleotide proportions are schematic and not drawn to scale. Abbreviations: ASO, antisense oligonucleotide; UBE3A-ATS, UBE3A antisense transcript; CNS, central nervous system; CSF, cerebrospinal fluid; mRNA, messenger RNA; DNA, deoxyribonucleic acid; RNA, ribonucleic acid; RNase H1, ribonuclease H1; NaV1.1, voltage-gated sodium channel subtype 1.1. Figure 1 was created in BioRender (https://BioRender.com/t6ab2h3).

Figure 1A contrasts the three strategies described in this subsection: productive-splicing enhancement, degradation of a protein-coding transcript and degradation of a repressive antisense transcript. Lowering a protein-coding transcript can reduce pathological activity when excessive function of its product drives disease. In an Scn2a gain-of-function mouse model, ASO-mediated transcript reduction improved seizures and survival, providing experimental support for this strategy. However, an ASO directed against a sequence shared by both alleles can reduce normal as well as mutant transcripts. Its pharmacological objective is a tolerable reduction in net pathogenic activity, rather than complete gene suppression. The distinction between gene-wide and allele-selective lowering therefore concerns both efficacy and preservation of physiological function [16].

Steric-blocking ASOs act through occupancy of a selected RNA sequence without recruiting RNase H1. Binding can prevent recognition of a splice site or regulatory element, thereby promoting exon inclusion, exon exclusion or avoidance of an aberrant splice event. The intended result depends on the function of the targeted element; exon skipping is not intrinsically corrective. Milasen illustrates mutation-specific splice repair: an ASO was designed to suppress abnormal processing of *MFSD8* RNA, and patient-derived assays showed improved splicing and lysosomal function; Section 4.6 describes the case in full. This establishes the importance of showing functional rescue downstream of an altered RNA product, rather than treating a change in splice pattern as sufficient evidence of benefit [17].

Targeted augmentation of nuclear gene output (TANGO) exploits endogenous non-productive splicing rather than a splice defect created by the pathogenic variant. Some normally transcribed pre-mRNAs undergo alternative splicing that introduces a premature termination codon and directs the resulting transcript toward nonsense-mediated decay. Blocking the relevant splice event can increase productive messenger RNA and full-length protein. Lim and colleagues demonstrated this approach across several targets in cellular and animal experiments. Its application requires a targetable regulatory event and an allele capable of producing useful protein. Augmentation cannot replace an absent coding sequence, and the presence of a non-productive transcript does not itself establish a therapeutic window [5]. In the *SCN1A* program, suppression of non-productive splicing increased productive transcript abundance and expression of the voltage-gated sodium channel NaV1.1 in experimental models, linking this pharmacological mechanism to the treatment of haploinsufficiency [8].

Reactivation of an epigenetically silenced allele uses a different route to protein augmentation. In neurons, expression of the paternal *UBE3A* allele is suppressed by the *UBE3A* antisense transcript (*UBE3A-ATS*). RNase H1-competent ASOs targeting this regulatory RNA can reduce *UBE3A-ATS* and relieve paternal silencing, thereby increasing expression from an available paternal allele. Meng and colleagues demonstrated sustained paternal *Ube3a* reactivation in cultured neurons and mice [6]. This mechanism combines degradation of one RNA with increased expression of another gene product. It must therefore be distinguished from non-degradative splice modulation, despite the shared objective of increasing protein abundance.

### 2.3. Delivery to the Central Nervous System and Productive Intracellular Availability

Central nervous system delivery imposes constraints that are independent of the choice of RNA mechanism. Figure 1B sets out the sequential requirements described in this subsection, from intrathecal administration to productive RNA engagement. Conventional unconjugated ASOs generally achieve insufficient brain exposure after systemic administration because their physicochemical properties restrict passage across the blood–brain barrier. Administration into cerebrospinal fluid, most commonly by lumbar intrathecal injection in clinical programs, provides access to the central nervous system [18]. However, bypassing the vascular barrier does not produce uniform parenchymal exposure. Multimodal imaging after intrathecal administration identified distribution along meningeal and vascular structures, subsequent tissue uptake and clearance into the systemic circulation. Drug concentration in lumbar cerebrospinal fluid therefore cannot be assumed to represent equivalent exposure across cortical, subcortical and spinal regions. This heterogeneity matters when the disease-relevant neuronal populations are distributed across several anatomical compartments [11].

Cellular uptake is an additional barrier to effective RNA modulation. Phosphorothioate ASOs can enter cells through endocytic pathways, but molecules retained within membrane-bound compartments cannot engage nuclear or cytoplasmic RNA. Productive delivery requires release from these compartments. In cellular experiments, interference with lysobisphosphatidic acid-dependent trafficking reduced antisense activity without reducing total cellular uptake, demonstrating that intracellular accumulation and functional availability are distinct measurements [19]. Consequently, tissue concentrations alone provide an incomplete account of pharmacological exposure. Evaluation must connect distribution with a mechanism-appropriate response, such as altered splice-isoform abundance, transcript reduction or restoration of the relevant protein.

The duration of ASO action reflects tissue retention, intracellular persistence and the turnover of the affected RNA and protein. Preclinical studies of the 2′-MOE-modified oligonucleotide subsequently developed as nusinersen demonstrated prolonged splice correction after central administration, including effects persisting for months in experimental animals [20]. Such observations support intermittent dosing but do not justify a universal dosing interval for the ASO class. A long tissue half-life may coexist with different rates of onset and recovery for different molecular targets. Dose selection should integrate exposure, target modulation, and tolerability for each ASO; administered dose, RNA modulation, and neuronal response are related but distinct measures.

### 2.4. Selectivity, Tolerability and the Therapeutic Window

Several properties favor ASOs as precision pharmacological interventions. They can act on endogenous RNA without delivery of an entire replacement gene, and their design can be adapted to a target sequence or a specific splicing abnormality. Endogenous protein augmentation may therefore be feasible without introducing an exogenous coding sequence [5,17]. Repeated administration also permits adjustment of subsequent dose levels or dosing intervals. This offers control over future exposure, although the degree and timing of reversibility remain compound-dependent. Discontinuation prevents further dosing but does not immediately remove tissue-retained drug or restore pre-treatment protein abundance. Prolonged action is therefore both an advantage for dosing frequency and a constraint when an adverse effect requires rapid reversal [18].

The molecular selectivity of an ASO must be established experimentally. Unintended effects can arise from excessive modulation of the intended target, hybridization to other transcripts, or interactions between the oligonucleotide and cellular proteins. The last mechanism is illustrated by studies in which toxic phosphorothioate gapmers altered protein localization and induced nucleolar stress; a restricted chemical modification reduced these effects and improved the therapeutic index in cellular and animal assays. These findings concern experimental chemistries and do not establish a uniform toxicity profile for intrathecal epilepsy treatments. They demonstrate that sequence and chemistry must be evaluated together [14]. Splice-switching ASOs also require assessment beyond their intended splice event. RNA sequencing in neuronally differentiated human induced pluripotent stem cells identified compound-dependent changes in splicing and gene expression. Only a minority of the differential splicing events could be explained by predicted hybridization. Sequence-based screening therefore benefits from experimental transcriptome analysis and validation of biologically relevant findings [21].

The therapeutic window additionally depends on how much target modulation the nervous system can tolerate. For transcript-lowering strategies, residual physiological expression may be essential; for augmentation strategies, increasing RNA is useful only if it generates functional protein at a tolerable level [5,16]. Repeated intrathecal procedures and longitudinal monitoring add a treatment burden that must be considered alongside molecular potency. These constraints favor an integrated assessment of productive delivery, functional rescue and tolerability. A favorable change in RNA abundance is an intermediate pharmacodynamic result; its therapeutic value depends on the biological consequences of that change in the relevant cells and at the treated stage of disease.

## 3. The Genotype-to-Mechanism Decision Axis

The selection of an ASO strategy requires a causal connection between the molecular diagnosis, the functional consequence of the variant and the intended change in gene output. Identifying a disease-associated gene does not establish whether its expression should be increased, reduced or redirected toward a different transcript. Molecular suitability must also be distinguished from clinical eligibility: a technically targetable variant may occur in a patient whose disease stage, affected tissues or treatment burden limit the expected benefit. Three questions guide candidate selection: in which direction gene output should be changed, how the functional effect and the relevant allele are established, and which outcomes may remain modifiable at the disease stage when treatment begins. Individualized-treatment frameworks therefore assess the variant, the disease, and the patient as distinct but interacting determinants of suitability [13].

### 3.1. Choosing the Direction of Intervention

In haploinsufficiency, the therapeutic objective is to increase functional protein from an available allele. *SCN1A*-related Dravet syndrome illustrates this approach when loss of function reduces effective NaV1.1 dosage. Enhancement of productive splicing can increase endogenous expression without introducing a replacement coding sequence. However, the relevant criterion is restoration of functional output, rather than an increase in total RNA alone. An augmentation strategy must retain a usable transcript and sufficient protein-coding capacity, and its effect on each expressed allele requires consideration. Increased expression of an abnormal protein cannot be assumed to be beneficial merely because the clinical phenotype includes epilepsy. The experimental basis for this approach comes from TANGO studies and ASO-mediated *Scn1a* augmentation in Dravet models [5,8].

Other forms of deficient protein expression require different solutions. In Angelman syndrome, reduction of *UBE3A-ATS* can release an available paternal *UBE3A* allele from neuronal silencing; suitability depends on the molecular configuration of the locus and the presence of a functional allele that can be reactivated [6]. For a pathogenic splice abnormality, the objective may instead be to restore productive processing of the affected allele. Milasen demonstrated the feasibility of this approach in *MFSD8*-related disease [17]. Neither example supports the general proposition that every loss-of-function disorder is amenable to ASOs. The actionable substrate is a specific RNA-regulatory mechanism or correctable processing defect.

When excessive activity of a gene product contributes to disease, transcript lowering may reduce the pathogenic drive. Experimental *SCN2A*, *SCN8A* and *KCNT1* programs support this rationale in selected gain-of-function models [16,22,23]. Gene-wide suppression nevertheless affects physiological expression as well as pathogenic activity. The required evidence concerns the range over which lowering improves function without producing an unacceptable deficit. A favorable result at one dose in one model cannot establish that complete suppression is desirable, or that the same exposure is appropriate for all variants within that gene. Dose-dependent functional rescue and preservation of normal activity are therefore joint requirements for candidate selection.

### 3.2. Establishing the Functional Effect and the Relevant Allele

Establishing the direction of intervention depends on knowing what the variant actually does, and not every functional deficit reduces to haploinsufficiency or to excess activity. Dominant-negative effects require separate analysis. A mutant product may impair the function of the wild-type product, particularly when both contribute to a multimeric complex. This can produce a functional deficit that differs from simple haploinsufficiency. In an electrophysiological study of seven encephalopathy-associated *KCNQ2* variants, five exerted marked dominant-negative effects on currents formed with wild-type K_v_7.2 or K_v_7.3 subunits [24]. The therapeutic implication is that indiscriminate transcript lowering could remove both the interfering product and the remaining source of normal function. Selective suppression of the mutant allele, augmentation of functional output or a combined strategy would require experimental comparison. These are mechanistic possibilities rather than established *KCNQ2*-directed ASO treatments. *KCNQ2* is included here to illustrate dominant-negative channel dysfunction and the need for variant-specific functional assessment; it is therefore not discussed as a separate therapeutic program in Section 4.

Allele-selective lowering separates pathogenic suppression from preservation of wild-type expression. Discrimination can exploit a sequence difference at the causal variant or another informative site on the same haplotype. The latter requires phasing to establish which sequence belongs to the pathogenic allele. Individualized *SCN2A* ASOs have used heterozygous intronic sequence differences, characterized through long-read sequencing, to preferentially reduce the mutant transcript in patient-derived neuronal models before clinical administration [25]. The relevant validation is quantitative: mutant and wild-type transcripts must be measured separately across a concentration range. Allele selectivity is a property to be demonstrated for a particular sequence and exposure, rather than inferred from the presence of a single mismatch.

Functional characterization must connect molecular measurements to the cellular phenotype. In *SCN2A*-related disorders, age at seizure onset and response to sodium-channel blockers can support a mechanistic hypothesis, but neither uniquely identifies the functional effect of a variant [3]. Channel assays may reveal changes in current density, voltage dependence, recovery from inactivation and persistent current that have different consequences for neuronal firing. Berecki and colleagues combined structural modeling, patch-clamp measurements and dynamic action potential clamp to investigate variants affecting fast inactivation of the voltage-gated sodium channel NaV1.2. For two non-inactivating variants, low current densities enhanced firing whereas higher densities produced sustained depolarization [26]. These findings show why an isolated biophysical change should not be equated automatically with a monotonic increase or decrease in neuronal activity. Variant interpretation should account for the expression system, channel density and complementary evidence at the neuronal level.

For suspected splice defects, RNA analysis addresses a different set of uncertainties: whether the variant changes processing, which isoforms are produced and whether correction restores a biologically useful product. Transcript analysis and functional assays are particularly important for deep intronic variants that may be missed by exon-focused sequencing. A systematic study in ataxia-telangiectasia provides the methodological precedent, linking genomic findings to validated splice correction and functional rescue. Its relevance here is methodological rather than evidential [27].

### 3.3. Timing and Disease Stage as Determinants of Therapeutic Opportunity

The third decision dimension is whether molecular correction can modify the outcomes of interest at the stage when treatment begins. Chronological age, disease duration and developmental status are related but distinct descriptors; none alone defines the potential for recovery. Three overlapping contributions warrant consideration: persistent neuronal dysfunction caused by altered gene-product activity, the effects of seizures and interictal epileptiform activity, and disturbances of circuit development that may be less amenable to later correction. These contributions interact, and developmental impairment independent of epilepsy should not be assumed to be irreversible. Conversely, seizure suppression does not by itself establish developmental recovery [1,28]. Developmental changes in target expression may further influence therapeutic opportunity, although prenatal enrichment alone does not establish a treatment window [29]. Candidate assessment should therefore distinguish prevention of emerging abnormalities, improvement of established manifestations, and slowing of further deterioration [13]. Preclinical studies support outcome-specific rather than uniform treatment windows. *Scn8a*-lowering ASOs provided benefit both after neonatal administration and when treatment began after seizure onset in mouse models of *SCN8A*-DEE [22,30]. Genetic reinstatement experiments in Angelman syndrome provide complementary mechanistic evidence: hippocampal plasticity remained recoverable after adult *Ube3a* reactivation, whereas motor coordination showed complete rescue after juvenile and partial rescue after adolescent reactivation; several other behavioral abnormalities required earlier restoration [31]. Adult reinstatement also restored prefrontal neuronal physiology despite previously reported limitations in behavioral recovery [32]. These genetic experiments should be distinguished from ASO treatment. With ASOs targeting *Ube3a-ATS*, administration on postnatal day 1 (P1) or postnatal day 21 (P21) rescued audiogenic seizure susceptibility, while P1 treatment produced selective behavioral improvements [33]. Separate ASO experiments demonstrated improvements in electroencephalographic (EEG) abnormalities and sleep disturbances in juvenile and adult mice [34]. These findings do not define corresponding age thresholds in humans, and differences in the extent and kinetics of molecular restoration complicate comparisons across approaches.

Clinical observations remain less conclusive. ASO treatment has been reported in a preterm infant with *SCN2A*-DEE and, at a later disease stage, in two approximately two-year-old children with *KCNT1* encephalopathy [10,35]. Open-label zorevunersen studies enrolled children and adolescents aged 2–18 years with established Dravet syndrome and reported seizure reductions alongside exploratory clinical and adaptive-function signals [9]. Such observations suggest that responses may remain possible after symptom onset, but do not establish reversal of developmental impairment. The clinical studies reviewed here do not establish the comparative benefit of initiating treatment at different disease stages.

Candidate development should therefore test interventions at clinically relevant stages and prespecify which outcomes are expected to remain modifiable, on what biological grounds and over what assessment period. Normalization of molecular or physiological readouts is not sufficient evidence of clinical recovery: in an inducible Angelman syndrome mouse model, *UBE3A* protein levels and hippocampal synaptic plasticity could be restored at ages when several behavioral phenotypes were no longer rescued [31]. Where a narrow treatment window is plausible, rapid molecular diagnosis becomes an integral part of the therapeutic pathway [36]. Developmentally appropriate outcome measures and credible comparators are needed to distinguish improvement, stabilization and slowed deterioration [37]. Disease stage should consequently inform candidate selection and interpretation of response alongside molecular mechanism and allele selectivity; diagnostic implementation is discussed in Section 6.2.

### 3.4. From Variant to Candidate: The Decision Framework

Candidate selection requires convergence between a credible molecular diagnosis, a defensible direction of intervention and a measurable functional response. Patient-specific considerations include the extent of potentially modifiable disease, accessibility of the relevant tissues and the feasibility of longitudinal assessment [13]. Table 1 summarizes the resulting decision framework. It is intended to organize the evidence required for translational development, rather than assign treatment on the basis of a gene name. Section 4 revisits the relevant molecular strategies in the context of clinical and preclinical programs, organized by gene rather than by mechanism. Figure 1 connects these molecular choices with delivery requirements and the distinction between target engagement, clinical benefit and modification of disease trajectory.

## 4. Translational Evidence, Disease by Disease

The programs below are grouped by target gene, so that each molecular situation set out in Section 3 can be followed through its own translational history. They are not of equal maturity: the available evidence ranges from rescue in mouse models alone to randomized controlled trials, and one phase 3 study has already reported a negative primary endpoint. Each subsection therefore states what the current data support and what they leave unresolved, and Table 2 collects those judgments program by program.

### 4.1. SCN1A and Dravet Syndrome: Zorevunersen and Splice-Mediated Augmentation

*SCN1A* augmentation addresses the reduced functional NaV1.1 dosage that characterizes most SCN1A-related Dravet syndrome. Zorevunersen, previously STK-001, increases productive splicing rather than replacing the gene. The preclinical program established a connection between increased *Scn1a* expression and suppression of disease manifestations: early intracerebroventricular administration reduced electrographic seizures and SUDEP in a Dravet mouse model [8]. The survival result strengthened the rationale for investigating outcomes beyond seizure counts; prevention of human SUDEP remains a separate question. Variant selection remains necessary because augmentation is intended for deficient functional output and cannot be extrapolated to every *SCN1A*-associated phenotype.

The published MONARCH and ADMIRAL phase 1/2a studies enrolled 81 patients aged 2 to 18 years; 75 entered open-label extensions. In participants initially receiving 70 mg, followed by extension doses up to 45 mg, median convulsive-seizure reductions ranged from 58.82% to 90.91% across monthly intervals during the first 20 extension months. Adaptive-behavior and quality-of-life improvements were also reported. These estimates describe a selected dose subgroup over multiple observation windows, not a randomized treatment effect. Post-lumbar puncture syndrome occurred in 25% during the initial studies, and elevated cerebrospinal-fluid protein in 45% during extensions. The report included suspected unexpected, serious adverse reactions in one participant and three deaths, two from SUDEP and one from malnutrition. These observations require safety interpretation without assuming drug causation [9].

The registered EMPEROR study uses a randomized, double-blind, sham-controlled phase 3 design [38]. A sponsor announcement reported completion of enrollment with 162 participants on 30 June 2026. Enrollment completion is a development milestone, not an efficacy result [39]. Controlled assessment can determine whether seizure and functional outcomes differ from those observed with continued standard care. Attribution of developmental benefit additionally requires attention to baseline trajectory, changes in background medication, and the distinction between raw-score gains and age-adjusted performance. The current evidence supports clinical development of a potentially disease-modifying therapy, while the magnitude and durability of developmental benefit remain to be established.

A separate *SCN1A*-directed clinical program is investigating ION337, a chemically distinct splice-modulating ASO designed to increase productive *SCN1A* expression and functional NaV1.1 availability [40]. The open-label phase 1/2 ASCEND study (NCT07531745) evaluates intrathecal ION337 in children aged 2–12 years with Dravet syndrome, with safety and tolerability as primary objectives and additional pharmacokinetic and pharmacodynamic assessments [41]. The sponsor reported first dosing in July 2026 [40]. No clinical efficacy or long-term safety conclusions can yet be drawn, and results from zorevunersen cannot be transferred to ION337 because the compounds and dosing strategies differ.

### 4.2. SCN2A: Elsunersen and Individualized Gapmers for Gain-of-Function Disease

*SCN2A*-directed lowering provides a mechanistic counterpart to *SCN1A* augmentation. In a gain-of-function *Scn2a* mouse model, ASO treatment reduced seizures and prolonged survival, establishing proof of concept for partial transcript suppression [16]. Translation depends on functional characterization because both deficient and excessive NaV1.2 activity can cause neurological disease. A gene-wide gapmer lowers transcripts from both alleles, whereas an allele-selective construct aims to reduce the pathogenic contribution while preserving wild-type expression.

Wagner and colleagues described elsunersen treatment in a preterm infant with a functionally confirmed *SCN2A* gain-of-function variant. Before treatment, status epilepticus had persisted for seven weeks, with approximately 20 to 25 seizures per hour. Nineteen intrathecal administrations over 20 months were associated with no reported severe or serious adverse events. In combination with sodium-channel blockers, status epilepticus was interrupted and subsequently ceased; seizure frequency decreased by more than 60%, although frequent seizures and severe developmental impairment persisted [10]. This experience supports feasibility and a possible antiseizure effect in severe disease. Concomitant treatment and the uncontrolled design prevent isolation of the ASO contribution or inference of developmental rescue. Kim-McManus and colleagues reported two prospective, individualized, allele-selective *SCN2A* ASO studies conducted as modified crossover N-of-1 trials in boys aged 9 and 14 years. Predefined observation periods were followed by ASO treatment and longitudinal follow-up within each participant; these were not randomized treatment-sequence crossover trials and included no pharmacological washout. Allele-selective constructs targeted informative sequence differences to preferentially reduce the pathogenic transcript. Reported seizure-frequency changes differed between participants (26% and 90%), and distinct variants, ASOs, baseline periods, ongoing development, concomitant treatment changes, and persistent ASO action limit causal attribution and preclude pooling the outcomes. Prospective within-patient measurement strengthens characterization relative to uncontrolled anecdotes, but does not yield an unbiased randomized efficacy estimate for the target population [25].

Controlled evidence has also been disclosed outside a peer-reviewed publication. In a sponsor communication dated 6 April 2026, EMBRAVE Part A, a randomized, sham-controlled phase 1/2 study involving nine children over a 24-week controlled period, was reported to achieve a 77% placebo-adjusted reduction in seizure frequency from baseline (95% confidence interval, 33–92%; *p* = 0.015) [42]. These preliminary findings have not yet been established in a full peer-reviewed clinical trial report. This small controlled study is distinct from both the infant report and the individualized allele-selective programs. Its interpretation requires the complete methods, participant-level disposition and prespecified analyses. A separate registered phase 3 elsunersen study uses a single-arm design [43]. Neither a positive sponsor disclosure nor a trial registration independently establishes modification of the developmental phenotype.

### 4.3. SCN8A: Preclinical Knockdown and Cross-Genotype Effects

*SCN8A* encodes the voltage-gated sodium channel NaV1.6, providing a target for reducing pathological excitability in selected gain-of-function disorders. Lenk and colleagues showed that an *Scn8a*-directed ASO delayed seizure onset and prolonged survival in a mouse model of *SCN8A* encephalopathy. The same intervention also improved survival in a Dravet model carrying an *Scn1a* defect [22]. The latter result is mechanistically informative: an intervention can reduce network excitability without correcting the primary molecular lesion. This supports investigation of shared physiological targets but does not imply that the underlying developmental consequences of different genetic disorders will respond equivalently.

Treatment after seizure onset is more relevant to most clinical diagnoses than exclusively presymptomatic rescue. Hill and colleagues subsequently evaluated repeated *Scn8a* ASO administration initiated after an observed convulsive seizure, with treatment repeated at four- to six-week intervals for up to one year in mice. The study supported prolonged benefit from sustained downregulation in established disease [30]. The experimental dosing interval cannot be transferred directly to children, and extended survival in mice does not resolve the effects of chronic NaV1.6 reduction on human motor, cognitive, or developmental function.

Together, these studies address complementary questions: whether *Scn8a* lowering can suppress a genetically driven epileptic phenotype, whether it remains effective after seizures emerge, and whether benefit can extend to another molecular etiology. The evidence discussed here is preclinical. Clinical translation requires a human-active sequence, an exposure range that preserves physiological channel function, and outcomes that separate antiseizure activity from changes in developmental course. Cross-genotype efficacy is therefore a rationale for further testing rather than a basis for assuming broad clinical applicability.

### 4.4. KCNT1: Knockdown and the Concept of Network-Node Lowering

*KCNT1* encodes the sodium-activated potassium channel K_Na_1.1, also called Slack. Although potassium conductances often restrain excitation, the consequences of *KCNT1* gain of function depend on neuronal subtype and firing dynamics; increased potassium current cannot be interpreted as uniformly protective. Burbano and colleagues tested *Kcnt1* lowering in a homozygous mouse model corresponding to a pathogenic human variant. Treatment of symptomatic animals reduced seizures, improved selected behavioral measures, and prolonged survival [23]. The model provides proof of concept, but its zygosity and experimental phenotype differ from the heterozygous disease usually encountered clinically.

*Kcnt1* suppression also prolonged survival in *Scn1a* and *Scn8a* mutant mice [44]. These findings support the concept of lowering a channel that influences pathological network activity across distinct initiating defects. Whether *KCNT1* is a broadly beneficial target, and whether cross-genotype seizure rescue reaches each disorder’s developmental pathology, both remain open. The distinction between intervention at the causal gene and modulation of a downstream network component remains relevant when interpreting disease-modifying claims.

Human evidence now extends beyond preclinical validation. Nakayama and colleagues reported intrathecal treatment of two 2-year-old girls with the recurrent *KCNT1* p.Arg474His variant using valeriasen, a non-allele-selective ASO. Seizure frequency and intensity decreased, but both patients developed ventricular enlargement or hydrocephalus. In patient 1, severe hydrocephalus prompted endoscopic third ventriculostomy with choroid plexus cauterization and subsequent withdrawal from the investigational protocol in favor of palliative care [35]. The report demonstrates that antiseizure improvement can coexist with a serious neurological safety signal. It does not establish a favorable overall benefit–risk balance, and the mechanism of the ventricular changes requires further investigation. Clinical tolerability cannot be inferred either from the absence of comparable toxicity in a disease model or from seizure reduction in an individual patient.

In the second patient, ventriculoperitoneal shunting was required. The investigators considered the ventricular complications attributable to study-drug dosing, although the underlying mechanism remained unresolved. Negative inflammatory investigations did not exclude these complications; subsequent lower-dose treatment in the surviving patient included ventricular imaging and cerebrospinal fluid (CSF) opening-pressure monitoring [35].

An additional *KCNT1*-directed investigational ASO, S230815, is being evaluated in the first-in-human, multicenter, open-label phase Ib/II KANDLE study (NCT07227857). The trial investigates the safety, tolerability, pharmacokinetics, and pharmacodynamic effects of repeated intrathecal administration in children aged 2–12 years with *KCNT1*-related developmental and epileptic encephalopathy [45]. Servier announced enrollment of the first participant in the United States on 25 June 2026, confirming that clinical development was underway before the review’s evidence cutoff [46]. No established clinical efficacy or long-term safety conclusions were available at the cutoff. S230815 should be distinguished from the individualized valeriasen program, and their benefit–risk profiles must be evaluated separately.

Developmental studies add another consideration. Golinski and colleagues identified functional *KCNT1*-associated conductance in prenatal human brain tissue and examined RNA-targeting effects in human neuronal preparations [29]. These findings suggest that the relevant physiology is already active before the usual postnatal treatment window. They support investigation of developmental timing but are not evidence that prenatal ASO administration is safe, effective, or clinically indicated. The translational problem encompasses both early target biology and the safety of altering that biology during brain development.

### 4.5. UBE3A and Angelman Syndrome: Allele Unsilencing

Angelman syndrome provides an alternative route to endogenous protein restoration because an available paternal *UBE3A* allele can be transcriptionally reactivated through targeting of *UBE3A-ATS*. Meng and colleagues established ASO-mediated paternal unsilencing in neuronal and animal systems [6]. Subsequent work in an Angelman mouse model showed restoration of UBE3A protein expression and rescue of several phenotypes, including susceptibility to audiogenic seizures, following early postnatal treatment [33]. These results connect molecular reinstatement with functional benefit, while the response of an induced seizure phenotype cannot establish the effect on spontaneous human epilepsy or the full neurodevelopmental syndrome.

The phase 1 TANGELO study evaluated rugonersen in 61 children aged 1 to 12 years using an open-label, multiple-ascending-dose design. The primary safety and tolerability objective was met. Exploratory analyses identified dose-dependent partial normalization of elevated electroencephalographic (EEG) delta power and signals of improvement in developmental and adaptive-function measures relative to natural-history expectations [47]. These findings support further investigation, but reduced delta power does not establish seizure reduction or cognitive benefit. Interpretation of natural-history comparisons also requires consideration of differences in age, molecular subtype, baseline ability and assessment procedures. EEG changes should therefore be interpreted as evidence of altered network physiology rather than as a validated surrogate for clinical efficacy.

Obudanersen (ION582) represents another investigational *UBE3A-ATS*-targeting ASO intended to increase paternal *UBE3A* expression [48]. In the open-label phase 1/2a HALOS study (NCT05127226) [49], sponsor-reported assessments indicated exploratory changes in communication, cognition, and motor function, with a reported tolerability profile supporting continued clinical development [48]. These findings were not established by randomized comparison and remain susceptible to maturation effects, measurement variability, and other confounding factors.

Obudanersen advanced to the randomized, double-blind, placebo-controlled phase 3 REVEAL study (NCT06914609) [50]. According to an Ionis announcement dated 6 July 2026, enrollment was completed in the pivotal pediatric cohort, comprising 136 participants aged 2 to under 18 years with Angelman syndrome associated with a UBE3A deletion or mutation; approximately 158 participants were planned across the pediatric and adult cohorts. The primary endpoint assesses expressive communication using the Bayley Scales of Infant and Toddler Development, Fourth Edition (Bayley-4); pivotal efficacy findings were not yet available at the review cutoff [51]. The separately registered, open-label, single-arm phase 3 CHAMPION study (NCT07782827) was designed for Angelman syndrome due to paternal uniparental disomy or imprinting defects, extending investigation to different molecular subgroups. Its registration was first posted on 24 August 2026 [52]. Although obudanersen received U.S. Food and Drug Administration (FDA) Breakthrough Therapy designation on 9 September 2025, this regulatory designation does not establish clinical efficacy or disease modification [53]. In particular, the negative Aspire results for apazunersen cannot be extrapolated to obudanersen, given differences between compounds, trial populations, regimens, and endpoints.

The apazunersen program illustrates the importance of controlled confirmation. Aspire was registered as a randomized, double-blind, sham-controlled phase 3 study [54]. On 2 September 2026, Ultragenyx reported that the study did not meet its primary endpoint, change in Bayley-4 cognitive raw score, or its key secondary endpoint, net response on the Multidomain Responder Index (MDRI). The sponsor described the safety profile as consistent with phase 1/2 experience [55]. This disclosure provides topline findings rather than a complete peer-reviewed trial analysis. The reported results do not confirm the developmental benefits suggested by earlier uncontrolled observations.

The negative Aspire result should be interpreted in relation to the tested compound, regimen, age range, genotype, follow-up duration, and endpoints rather than interpreted as evidence that targeting UBE3A-ATS cannot produce clinically meaningful developmental benefits. The study was restricted to children aged 4–17 years with a full maternal deletion, a genotype affecting additional genes beyond *UBE3A*. Potential outcome-specific developmental windows may restrict reversibility after later intervention. The dosing and administration strategy had been modified after early lower-extremity weakness reports, but whether the eventual regimen provided sufficient CNS exposure and target engagement remains uncertain. Bayley-4 cognitive raw scores over 48 weeks in an age range extending beyond the scale’s standard normative population warrant careful interpretation; nevertheless, the key secondary multidomain responder index also failed. These factors are hypotheses for interpretation, not established explanations of the negative trial, and neither negate the controlled result nor demonstrate efficacy of other compounds [56,57,58].

Safety interpretation must also account for earlier experience with apazunersen, then designated GTX-102. A sponsor report dated 26 October 2020 described lower-extremity weakness classified as a serious adverse event in all five initially treated participants: four after the highest dose and one after the second-highest dose. These events prompted suspension of dosing and enrollment, with planned changes to dose levels and administration. Local inflammation was proposed as an explanation, supported by reported imaging findings, but the report did not establish a compressive mass effect [59]. This observation should not be generalized to every ASO targeting *UBE3A-ATS.* Across Angelman programs, molecular reactivation, EEG changes, neurodevelopmental outcomes and seizure outcomes should be assessed separately. Mechanistic plausibility and preclinical rescue provide a rationale for clinical testing; demonstration of patient benefit requires controlled evidence for the specific compound, regimen and population studied.

### 4.6. MFSD8 and Individualized N-of-1 Development: Milasen and Subsequent Frameworks

Milasen established the feasibility of developing an ASO for a private pathogenic splice defect in a child with *MFSD8*-related neuronal ceroid lipofuscinosis type 7 (CLN7). Genomic and RNA analyses identified abnormal splicing associated with an intronic insertion; a patient-specific ASO improved processing and lysosomal function in cellular assays before intrathecal administration. In comparisons of serial electroencephalographic recordings, both seizure frequency and mean seizure duration decreased by more than 50%, and the percentage of recording time spent in seizures decreased by more than 80% [17]. These estimates arose from a single uncontrolled treatment program and limited recording windows. They establish an observed antiseizure signal rather than a generalizable effect size. Because CLN7 disease is neurodegenerative, the case also demonstrates why seizure improvement must be distinguished from arrest or reversal of the underlying disorder.

The subsequent development of systematic splice-switching frameworks has made candidate assessment more reproducible. Kim and colleagues combined genomic analysis, prediction of splice amenability and cellular validation to identify potentially treatable variants in ataxia-telangiectasia [27]. Although this is a different disease, the workflow is relevant to epilepsy-associated splice variants: the molecular defect must be demonstrated, the intended transcript restored and downstream function assessed. A sequence that changes RNA processing is not automatically a clinically suitable medicine. Individualized *SCN2A* studies further show that N-of-1 development can include allele-selective transcript lowering, rather than being restricted to splice correction [25].

Consensus recommendations for N-of-1 exon-skipping ASOs address oligonucleotide design and in vitro efficacy testing, while broader patient-evaluation frameworks integrate molecular amenability with disease course and individual circumstances [13,60]. These documents support consistent evaluation and data sharing, but do not replace compound-specific toxicology, clinical oversight or prospective outcome assessment. Small populations increase the value of predefined measures, stable observation periods where feasible and explicit documentation of concomitant treatment. The scientific contribution of an individualized program includes both its patient-level outcome and the reproducibility of the evidence linking variant, intervention and response.

### 4.7. GNAO1: Emerging Allele-Selective ASO Development

The registered open-label phase 1/2 tianasen (ASO-GNAO1) study (NCT07363603) investigates repeated intrathecal administration in a small, variant-defined population with *GNAO1*-related encephalopathy, epilepsy, and movement disorders [61]. The investigational strategy aims to preferentially reduce expression of the mutant *GNAO1* transcript associated with c.607G>A (p.Gly203Arg). At the evidence cutoff, the registration established clinical investigation, not efficacy or long-term safety. Separately, preclinical work on allele-selective *GNAO1* ASOs provides a mechanistic precedent, but was conducted in a different pathogenic variant and should not be treated as evidence for the tianasen compound [62].

Table 2 distinguishes the principal evidence levels across these programs and presents distinct clinical compounds and study designs in separate rows. Shared use of an ASO platform does not make results interchangeable: molecular direction, allele selectivity, disease stage, administration schedule and study design all influence the inference that can be drawn.

**Table 2:** Translationalevidence and principal limitations of ASO programs in genetic epilepsies.

Target or Program	Evidence Category/Reporting Status	Principal Evidence	Supported Inference	Reported Safety Findings	Principal Limitation
*SCN1A*/zorevunersen	Published preclinical studies; published uncontrolled phase 1/2 studies; registered controlled phase 3 (no outcomes)	Mouse rescue studies [8]; published phase 1/2a studies and open-label extensions [9]; registered sham-controlled phase 3 EMPEROR study [38], with sponsor-reported enrollment completion [39].	Preclinical efficacy and preliminary human clinical signals support controlled evaluation.	Post-lumbar puncture syndrome (25%, initial studies); elevated CSF protein (45%, extensions). Serious events and deaths were reported; attribution requires separate assessment.	Developmental efficacy and human SUDEP reduction remain unconfirmed.
*SCN1A*/ION337 (ASCEND)	Registered open-label early-phase trial; no efficacy findings	Registered open-label phase 1/2 dose-escalation study; first participant dosed in 2026 [40,41].	Clinical investigation of another splice-modulating *SCN1A* ASO.	No clinical safety outcomes publicly reported at the cutoff.	No controlled efficacy or long-term safety evidence; distinct from zorevunersen.
*SCN2A */elsunersen (individual treatment)	Published single-patient treatment	Published treatment of one preterm infant over 20 months [10].	Feasibility and a possible antiseizure signal during combined treatment.	No serious adverse events reported during the published single-patient treatment involving 19 intrathecal administrations.	Uncontrolled observation with concomitant sodium-channel blockers; severe developmental impairment persisted.
*SCN2A */elsunersen (EMBRAVE Part A)	Randomized controlled phase 1/2; preliminary sponsor disclosure	Sponsor-reported randomized, sham-controlled phase 1/2 findings: nine children, 24-week controlled period; disclosure dated 6 April 2026 [42].	The reported sham-adjusted seizure reduction supports further evaluation.	No drug-related serious adverse events or discontinuations disclosed by the sponsor; full safety analysis unavailable.	Small sample and sponsor-reported topline results; complete methods and efficacy and safety analyses are needed for full appraisal.
SCN2A/individualized allele-selective ASOs	Published prospective modified-crossover N-of-1 studies (two patients)	Published prospective modified-crossover N-of-1 studies in two boys using distinct allele-selective ASOs [25].	Feasibility of individualized allele-selective treatment, with heterogeneous antiseizure signals.	No ASO-related serious adverse events reported in the two published individualized studies.	No controlled efficacy estimate; different variants, constructs and concurrent treatment changes limit comparison and attribution.
*SCN8A* lowering	Published preclinical evidence only	Preventive and post-onset ASO treatment in mouse models [22,30].	Preclinical antiseizure and survival benefits support further translational investigation.	Human safety not established; preclinical evidence only.	These experiments do not establish the human benefit–risk balance or the optimal degree of *SCN8A* reduction.
*KCNT1*/valeriasen	Published preclinical studies and two-patient clinical report	Mouse studies using experimental *Kcnt1*-targeting ASOs [23]; published valeriasen treatment in two patients [35].	Preclinical efficacy and clinical antiseizure signals are accompanied by an important safety concern.	Ventricular enlargement or hydrocephalus occurred in both treated patients, requiring neurosurgical intervention.	Ventricular enlargement or hydrocephalus was reported in both treated patients; uncontrolled observations do not establish net clinical benefit.
*KCNT1 */S230815 (KANDLE)	Registered open-label early-phase trial; no efficacy findings	Registered first-in-human, open-label phase Ib/II KANDLE study [45,46].	Clinical evaluation of a distinct intrathecal *KCNT1*-targeting ASO.	No clinical safety outcomes publicly reported at the cutoff.	No established efficacy or long-term safety data; results cannot be inferred from valeriasen.
*UBE3A-ATS*/rugonersen	Published uncontrolled clinical study	Published open-label phase 1 TANGELO study involving 61 children [47].	Exploratory EEG and developmental findings support controlled evaluation.	Pyrexia and vomiting reported; serious adverse events occurred, including treatment-related events.	No randomized comparator; natural-history comparisons are vulnerable to confounding, and EEG delta-power changes are not a validated surrogate for clinical benefit.
*UBE3A-ATS/obudanersen (ION582)*	Sponsor-reported uncontrolled findings; registered phase 3 trials without reported efficacy outcomes	HALOS phase 1/2a sponsor-reported findings [48,49]; randomized phase 3 REVEAL study [50,51]; registered single-arm phase 3 CHAMPION study [52].	Sponsor-reported exploratory functional signals support further evaluation; controlled efficacy results were not available at the evidence cutoff.	Favorable preliminary tolerability reported by the sponsor for HALOS; complete published safety data unavailable.	HALOS uncontrolled and sponsor-reported; pivotal efficacy and long-term safety not established.
*UBE3A-ATS */apazunersen (Aspire)	Controlled phase 3 trial; sponsor-reported negative topline findings	Registered randomized, double-blind, sham-controlled phase 3 Aspire trial [54]; negative topline findings disclosed on 2 September 2026 [55].	The trial did not demonstrate benefit on the prespecified primary cognitive or key secondary multidomain responder endpoint for the tested regimen and population.	Lower-extremity weakness reported in five early phase 1/2 participants (2020) [59]; Aspire safety described by the sponsor as consistent with earlier experience, without detailed event rates [55].	Sponsor-reported topline findings; restriction to children aged 4–17 years with full maternal deletions limits generalizability. Uncertainties regarding developmental timing, modified dosing, and endpoint sensitivity do not negate the negative primary and key secondary results.
*MFSD8 */milasen	Published single-patient treatment	Individualized splice correction and single-patient treatment [17].	Feasibility of variant-specific ASO development and an observed antiseizure signal.	No serious adverse events reported in the published single-patient treatment.	Uncontrolled observation does not establish arrest of neurodegeneration or generalizable efficacy.
*GNAO1/tianasen (ASO-GNAO1)*	Registered early-phase clinical study; no efficacy findings	Registered open-label phase 1/2, c.607G>A variant-defined study [61].	Early clinical evaluation of allele-selective *GNAO1* RNA lowering.	No clinical safety outcomes publicly reported at the cutoff.	Very small selected population; no controlled efficacy estimate or established long-term safety.

Evidencecategories are not directly interchangeable: preclinical experiments, individual treatments, uncontrolled studies, controlled trials and sponsor disclosures support different levels of inference. Trial registration documents study design, not efficacy. Murine ASOs and clinical compounds listed under the same gene target are not necessarily identical. Milasen is included as a translational precedent in a genetic neurodegenerative disorder with epilepsy. Safety findings are reported according to the original source and the 28 September 2026, evidence cutoff. Absence of reported serious adverse events does not establish long-term safety, particularly in small or uncontrolled studies. Sponsor-reported findings are not equivalent to complete peer-reviewed analyses. No reported safety outcomes indicate that clinical results were unavailable rather than an absence of adverse events. Abbreviations: ASO, antisense oligonucleotide; CSF, cerebrospinal fluid; EEG, electroencephalography; SUDEP, sudden unexpected death in epilepsy; UBE3A-ATS, UBE3A antisense transcript.

## 5. Safety, Tolerability and the Burden of Intrathecal Delivery

### 5.1. Hazards Attributable to the Compound

The safety of an ASO depends on its sequence, chemical architecture, intended molecular effect and tissue exposure, together with the risks of its administration. Hybridization to unintended transcripts can alter RNA processing or abundance, whereas phosphorothioate-dependent protein interactions can produce toxicity independently of sequence complementarity. Experimental studies demonstrate that chemical modification can reduce abnormal protein binding and cellular toxicity, and that splice-switching ASOs require empirical assessment of unintended splicing events [14,21]. A separate concern is excessive on-target activity: non-allele-selective knockdown reduces both mutant and wild-type transcripts. The *SCN2A* mouse model, in which partial transcript suppression reduced spontaneous seizures and extended survival while behavioral performance remained comparable to wild-type animals, illustrates why maximal suppression is not an appropriate universal objective [16]. Safety assessment must therefore accompany optimization of molecular efficacy, rather than follow from it.

Cerebrospinal fluid (CSF) abnormalities require interpretation within each treatment program. In the zorevunersen studies, elevated CSF protein was the most frequently reported adverse event in the extensions, affecting 45% of participants; post-lumbar puncture syndrome occurred in 25% during the initial phase 1/2a studies [9]. These observations do not establish that CSF protein elevation or pleocytosis is invariably transient or clinically inconsequential. Relevant experience comes from tofersen, although its recipients were adults with SOD1-associated amyotrophic lateral sclerosis, rather than children with epilepsy. A pooled clinical safety analysis identified 12 serious neurological events in 10 of 147 participants exposed to 100 mg: myelitis, radiculitis, aseptic meningitis and intracranial hypertension or papilledema. Most events resolved, but some required treatment interruption or discontinuation [63]. These findings identify possible complications of central ASO treatment without supplying incidence estimates for epilepsy-directed compounds. The valeriasen experience illustrates that clinically significant neurological complications cannot be excluded solely by reassuring routine CSF inflammatory findings. Neurological surveillance during dose escalation should therefore be informed by symptoms and, when indicated, neuroimaging and CSF pressure measurements. This compound-specific safety signal must not be extrapolated as a uniform risk across ASO programs [35]. Early apazunersen experience highlights the need for compound- and regimen-specific neurological monitoring during dose escalation. Proposed mechanisms of neurological toxicity, especially when derived from sponsor disclosures, should not be described as established without corroborating evidence. The observed safety signal cannot be assumed to apply uniformly to all *UBE3A-ATS*-directed ASOs [59].

Systemic hazards also require compound-specific interpretation. Current nusinersen prescribing information advises platelet counts, coagulation testing, and quantitative urine protein measurement before treatment and each dose because of bleeding and renal safety concerns [64]. This provides a regulatory precedent for surveillance, not an estimate of risk for investigational epilepsy ASOs. Complement activation is similarly unsuitable for unqualified extrapolation. An integrated analysis of 12 systemically administered 2′-O-methoxyethyl ASOs found that nonhuman primates overpredicted complement activation and platelet effects in healthy human volunteers [65]. Chemistry, route, exposure, and species therefore matter when translating toxicological findings into clinical monitoring requirements.

Pharmacological persistence creates a trade-off between dosing convenience and control of adverse effects. ASOs permit adjustment of subsequent doses and do not require permanent genomic alteration, but discontinuation does not immediately terminate target modulation. Preclinical nusinersen studies demonstrated sustained splice correction for months after central administration [20]. The rate of recovery after withdrawal depends on tissue drug clearance and restoration of the affected RNA and protein pools. Moreover, reversibility of target modulation does not guarantee reversal of an established neurological injury. Dose escalation should therefore account for delayed pharmacodynamic effects and accumulation during repeated administration; the absence of acute intolerance does not establish the safety of a maintenance regimen.

### 5.2. Hazards and Burden Attributable to Administration

Intrathecal administration also produces spatially heterogeneous exposure. Preclinical imaging demonstrated rostral distribution, meningeal association, tissue uptake and partial peripheral clearance after lumbar injection [11]. Thus, increasing a dose to improve engagement in relatively inaccessible brain regions may also increase exposure elsewhere. CSF drug concentration alone cannot establish the magnitude or uniformity of intracellular target modulation. The delivery sequence illustrated in Figure 1B is relevant to safety as well as efficacy: administration into CSF, regional distribution and productive cellular uptake are separate determinants of the eventual pharmacological effect.

Repeated lumbar puncture adds risks and burdens independent of ASO chemistry. In an early nusinersen study involving 28 children with spinal muscular atrophy, adverse events attributed to lumbar puncture followed 23 of 73 procedures (32%), most commonly headache, back pain and post-lumbar puncture syndrome [66]. These procedure-based estimates should not be directly compared with participant-based adverse-event rates in epilepsy trials. Positioning, analgesia, sedation and image guidance require individual assessment, particularly in children with motor impairment, spinal deformity or limited ability to cooperate. Experience in spinal muscular atrophy establishes feasibility, but the relevant respiratory, behavioral and seizure-related vulnerabilities differ across DEEs.

For children requiring repeated treatment, the burden extends beyond the injection. Hospital attendance, travel, fasting when anesthesia is required, recovery time and disruption of schooling or rehabilitation should be measured alongside clinical outcomes. In epilepsy protocols, procedural planning should also preserve scheduled antiseizure treatment and provide an individualized rescue plan. These are practical considerations for trial and service design, rather than evidence that a particular ASO increases peri-procedural seizure risk. Recording failed punctures, sedation exposure, unplanned admissions and caregiver time would make the burden of administration more transparent than reporting completed doses alone.

### 5.3. Surveillance and Alternative Routes of Access

The available safety signals support prospective, protocol-specific surveillance. Baseline and serial neurological assessment, systematic symptom recording and longitudinal interpretation of CSF findings can help distinguish disease fluctuation, procedural complications and drug-associated toxicity. New weakness, persistent headache, vomiting, altered alertness or visual symptoms warrant assessment rather than automatic attribution to the underlying disorder. The valeriasen and tofersen experiences support targeted investigation of CSF circulation and neuroinflammation when clinically indicated [35,63]. The intensity of imaging, ophthalmological assessment, and laboratory monitoring should reflect the compound and observed signal; a validated universal surveillance schedule for epilepsy ASOs has not been established.

Implantable access devices may reduce repeated puncture requirements but introduce their own complications. In a prospective nusinersen study of 17 participants with complex spinal anatomy, four subcutaneous intrathecal catheter systems required surgical revision and one was removed following Staphylococcus epidermidis meningitis [67]. These data support selective investigation of alternative access, rather than routine substitution for lumbar puncture in DEEs. Long-term acceptability will depend on the combined burden of the drug, the access procedure and monitoring. Demonstrating seizure reduction is therefore insufficient for a favorable benefit–risk assessment unless its magnitude and durability justify these cumulative demands and any treatment-associated neurological injury.

## 6. Challenges and Future Directions

### 6.1. Delivery and Durability of Target Modulation

Improved delivery must increase productive exposure in disease-relevant cells while reducing the burden of repeated central administration. Implantable reservoirs address access but do not, by themselves, correct heterogeneous parenchymal distribution or inefficient intracellular trafficking. Blood–brain barrier transport offers a different approach. Barker and colleagues conjugated a tool ASO to a transferrin receptor-binding transport vehicle, achieving widespread RNA knockdown after systemic administration in humanized mice and nonhuman primates. In macaques, this approach produced more uniform CNS distribution and target suppression than intrathecal delivery of the unconjugated ASO [68]. The target was *MALAT1*, rather than an epilepsy-associated transcript. Translation therefore requires demonstration of efficacy, cell-specific pharmacology and tolerability for the intended therapeutic sequence, including the consequences of peripheral exposure [68].

Durability can also be increased through vector-encoded RNA silencing, although this changes the therapeutic modality. In *SCN8A* mouse models, repeated ASO dosing after seizure onset and neonatal administration of an adeno-associated virus (AAV)-delivered short hairpin RNA both provided long-term protection in separate experimental arms [30]. These findings support sustained target reduction but do not establish equivalence between the approaches. Vector-expressed RNA interference differs from synthetic ASO pharmacology and offers less direct control over treatment withdrawal. Development should compare the required magnitude and duration of gene modulation, anatomical coverage and manageability of adverse effects, rather than regard longer persistence as an unconditional advantage.

### 6.2. Timing of Intervention and the Diagnostic Pathway

As discussed in Section 3.3, preclinical evidence supports outcome-specific therapeutic windows but does not establish corresponding age thresholds for patients [34]; related post-onset experiments further illustrate ongoing seizure-network responsiveness [30]. Clinical studies should distinguish chronological age, disease duration, baseline abilities, and the specific developmental process being assessed.

Earlier treatment requires a diagnostic pathway that operates on a clinically relevant timescale. In the Gene-STEPS pilot cohort, rapid genome sequencing identified a genetic diagnosis in 43 of 100 infants with new-onset epilepsy, with a median interval of 21 days from enrollment to result [36]. Molecular diagnosis must then be connected to functional interpretation, ASO eligibility and access to treatment. Presymptomatic newborn genomic screening could eventually shorten this pathway further, but would require evidence concerning penetrance, predictive accuracy and the benefit–risk balance of treating an infant before symptoms. Prenatal diagnosis must also be distinguished from prenatal drug administration, for which these postnatal studies provide no clinical efficacy or safety evidence.

### 6.3. Trial Design, Rare-Variant Designs and Biomarkers

Trial design must accommodate small populations without weakening causal inference. Eligibility should reflect the relevant syndrome, molecular mechanism and disease stage, consistent with the proposed operational framework for DEE trials [28]. Concurrent randomized controls remain valuable because seizure frequency fluctuates and developmental outcomes change with age, concomitant medication and supportive interventions. Where blinding requires a sham procedure, its components should be justified individually: maintaining masking does not automatically require dural puncture or intrathecal placebo administration. Rescue treatment, exposure duration and access after trial completion require explicit consideration, particularly when uncontrolled seizures carry substantial morbidity.

For variants too rare to support conventional trials, prospective within-patient baselines and carefully aligned natural-history cohorts can strengthen interpretation. Their limitations remain substantial: regression to the mean, ascertainment differences, unequal follow-up and changes in background care can imitate treatment effects. Eligibility criteria, outcome definitions, assessment schedules and handling of missing observations should be specified before comparison. Adaptive or Bayesian approaches may improve efficiency but cannot remove bias from an inappropriate comparator. Conventional crossover N-of-1 designs are also difficult to apply to persistent ASO effects: prolonged carryover and ongoing development can prevent a return to a comparable untreated state. Individualized treatment therefore requires individualized evidence collection, rather than an assumption that every patient can serve as an adequate control [13].

Biomarkers should distinguish drug exposure, molecular target engagement, network response and clinically meaningful benefit, as represented in Figure 1C. The partial normalization of EEG abnormalities observed with rugonersen supports quantitative electrophysiology as a pharmacodynamic measure [47]. It does not validate EEG change as a surrogate for developmental improvement or seizure reduction. Biomarker analyses should account for age, genotype, vigilance state, recent seizures and sedating medication. Similarly, a proposed CSF RNA or protein assay requires analytical validation and evidence that its signal reflects the relevant neuronal process. Longitudinal association with clinical change is necessary but does not alone prove that a biomarker can replace a clinical endpoint.

### 6.4. Expanding the Target Space

Greater molecular precision depends on scalable assessment of variant function and ASO susceptibility. The *N*= 1 Collaborative (N1C) VARIANT consensus provides a structuredapproach to evaluating eligibility for splice correction, exon skipping and transcript downregulation, while recognizing the additional uncertainty surrounding upregulation strategies [69]. Eligibility is a starting point for development, not evidence of clinical benefit. Allele-selective ASOs could preserve physiological expression while suppressing a pathogenic transcript, as illustrated by the individualized *SCN2A* programs [25]. Such approaches require correct haplotype phasing and a sequence that discriminates between alleles. The frequency of compatible haplotypes, including variation across ancestry groups, should be assessed before extrapolating a successful individual design to a wider population.

Human neuronal models can identify therapeutic opportunities that are poorly represented in conventional animals. In Timothy syndrome, ASOs reduced inclusion of the developmentally regulated *CACNA1C* exon 8A and corrected selected cellular abnormalities in patient-derived neurons, organoids and assembloids, with additional validation in transplanted human neural tissue [70]. This provides preclinical evidence for isoform modulation rather than indiscriminate transcript suppression. It does not establish clinical seizure efficacy, and a CNS-directed intervention would require separate assessment of the syndrome’s extracerebral manifestations. Such studies are most informative when correction of RNA processing is linked to protein function and a disease-relevant cellular phenotype.

*SYNGAP1* provides another example of expansion beyond the initial ion-channel targets. Disrupting polypyrimidine tract-binding protein 2 (PTBP2) binding to *SYNGAP1* pre-mRNA can redirect non-productive splicing and increase expression in patient-derived neurons [71]. More recently, humanized SYNGAP1 models supported human-sequence ASO testing in derived neurons and reproduced EEG abnormalities with parallels in patients; the same study developed STXBP1 humanized or hybrid models for therapeutic investigation [72]. These are experimental tools and molecular proof-of-concept findings, not evidence of ASO-mediated clinical rescue. Proposed applications involving STXBP1 or *CDKL5* still require a defined RNA substrate, restoration of functional protein and demonstration that the intervention preserves appropriate dosage and cellular regulation. For *KCNQ2* variants with dominant-negative effects, target reduction cannot be selected from the gene name alone, because channel function depends on the consequences for both mutant and normal subunits [24].

Combination treatment should be evaluated as a pharmacological question. In the reported *SCN2A* infant, elsunersen was administered alongside sodium-channel-blocking antiseizure medications, preventing attribution of the observed response to ASO monotherapy [10]. Combining correction of transcript abundance with modulation of channel activity may be useful but could also produce excessive functional suppression. Trials should document background treatment, stabilize it when feasible and prespecify how dose changes will be incorporated into analysis. Reductions in sedating medication may themselves improve alertness and performance; these benefits remain clinically relevant, but complicate attribution of developmental gains to direct molecular rescue.

### 6.5. Access and Infrastructure

Access depends on more than the cost of synthesizing an oligonucleotide. Diagnostic testing, functional studies, sequence selection, toxicology, quality-controlled manufacture, regulatory preparation, administration and long-term follow-up each require specialized capacity. The N = 1 Collaborative has organized shared work on these components, including data coordination and access [73]. Common protocols and reusable manufacturing processes could reduce duplication, but a new sequence still requires appropriate validation. Sustainable programs must also specify responsibility for continued supply, safety surveillance and clinical care after initial investigational funding ends. Selection should be transparent and clinically justified, with access to evaluation extending beyond families able to finance development or reach a small number of specialist centers.

Individualized N-of-1 ASO development also raises ethical and regulatory challenges beyond technical feasibility. The distinction between experimental treatment and clinical research should be clearly communicated to patients and families. For pediatric patients, informed permission from legally authorized representatives should address uncertain clinical benefits, potentially prolonged toxicity, the burden of intrathecal administration, and limitations in predicting functional recovery; developmentally appropriate assent should be sought when feasible [74]. Independent ethical review and regulatory oversight should follow the applicable jurisdictional framework. In the United States, FDA draft guidance provides nonbinding recommendations for individualized ASO development, whereas European regulatory pathways require separate consideration [75,76]. Multidisciplinary benefit–risk assessment, transparent patient selection, and responsible data sharing are essential to support equitable access and accountable clinical development [13,73].

### 6.6. Outcome Standards and the Evidentiary Threshold

The evidentiary standard must ultimately encompass the features that determine patients’ daily lives. The international consensus on outcomes in pediatric epilepsy identifies behavioral, motor and cognitive or language development as essential domains alongside seizure-related outcomes [37]. For ASO trials, these domains should be selected prospectively, measured with tools appropriate to the child’s abilities and analyzed with attention to multiplicity, floor effects and meaningful change. They need not all be designated primary endpoints but should have sufficient methodological support to test claims extending beyond seizure suppression. Raw developmental gains, age-standardized performance and maintenance of previously acquired skills describe different outcomes and should not be treated as interchangeable. A claim of developmental benefit should (i) specify the domain, instrument, assessment interval and clinically meaningful threshold in advance; (ii) compare change with a credible concurrent control or an appropriately aligned natural-history trajectory; and (iii) report background medication changes, missing data and uncertainty. A higher raw score during maturation alone is not evidence of an altered developmental trajectory.

Mortality and SUDEP require similarly disciplined assessment. Reduced seizure frequency cannot establish a survival benefit, and small early-phase trials are unlikely to estimate changes in rare fatal outcomes reliably. Prospective event adjudication, adequate exposure denominators and long-term follow-up across studies are needed. More broadly, evidence of disease modification should demonstrate a sustained alteration in clinically relevant trajectories, supported by an appropriate comparator and interpreted alongside molecular and physiological findings. No single biomarker, caregiver impression or short-term developmental score can establish modification of the whole encephalopathic phenotype. The objective is a coherent pattern of benefit across prespecified domains, with its magnitude, uncertainty and treatment burden made explicit.

## 7. Conclusions

Antisense oligonucleotides provide a pharmacological means of addressing selected molecular causes of genetic epilepsies and developmental and epileptic encephalopathies. Their therapeutic direction depends on the functional consequence of the variant: increasing productive gene output in haploinsufficiency, reducing pathogenic transcripts in appropriate gain-of-function disorders, correcting abnormal splicing or reactivating a silenced allele. These strategies require preservation of physiological protein function and cannot be assigned solely from a gene name. Functional characterization and demonstrable RNA amenability are therefore prerequisites for development, rather than guarantees of therapeutic success [13,69].

The clinical evidence supports feasibility and provides signals of benefit, but its strength differs substantially across compounds and indications. Open-label zorevunersen studies reported seizure reductions and exploratory improvements in adaptive function, supporting controlled evaluation of its disease-modifying potential [9]. Conversely, the coexistence of seizure improvement and hydrocephalus during *KCNT1*-directed treatment demonstrates that molecularly rational intervention can carry substantial neurological risk [35]. According to sponsor-reported topline findings, the randomized phase 3 Aspire study of apazunersen in Angelman syndrome failed to meet its primary Bayley-4 cognitive raw-score endpoint and its key secondary Multidomain Responder Index endpoint [55]. Although the absence of a complete peer-reviewed analysis limits detailed appraisal, the negative controlled findings do not confirm the developmental benefits suggested by earlier uncontrolled observations.

Further progress requires reliable CNS delivery, appropriate control of target dosage, identification of outcome-specific treatment windows and sustainable access to molecular diagnosis and treatment. Dose adjustability remains useful, but prolonged tissue activity limits the speed with which an unwanted effect can be withdrawn. Clinical studies must combine credible comparators with prospective developmental assessment and long-term safety follow-up, accounting for concomitant treatment and the cumulative burden of intrathecal administration. Ultimately, the clinical value of ASOs will depend on durable, patient-relevant benefits that justify the associated risks and treatment burden.

## Figures and Tables

**Table 1:** Variant-to-intervention framework for ASO development in genetic epilepsies.

Molecular Situation	Intended Direction of Intervention	Illustrative Application	Principal Validation Requirement
Haploinsufficiency with a targetable non-productive splice event	Increase productive transcript and functional protein	*SCN1A* augmentation [5,8]	Demonstrate useful protein output and assess the consequences of increasing expression from each available allele.
Gain of function with a tolerable reduction window	Partially lower the protein-coding transcript	Selected *SCN2A*, *SCN8A* and *KCNT1* models [16,22,23]	Establish functional rescue across doses while preserving sufficient physiological activity.
Pathogenic allele distinguishable by an informative sequence	Preferentially lower the pathogenic transcript	Individualized *SCN2A* ASOs [25]	Establish haplotype phase and measure suppression of both alleles separately.
Functional allele subject to regulatory silencing	Target the repressive antisense transcript	*UBE3A-ATS* lowering in Angelman syndrome [6]	Confirm a reactivatable allele and appropriate restoration of expression.
Pathogenic abnormal splicing	Redirect processing towards a useful transcript	*MFSD8* splice correction with milasen [17]	Demonstrate correction of the RNA defect and downstream functional rescue.
Dominant-negative effect or unresolved functional mechanism	Compare mechanism-specific strategies before selecting augmentation or suppression	*KCNQ2* as an illustration of dominant-negative channel dysfunction [24]	Establish how changing mutant and wild-type expression affects net function; an ASO strategy is not established by the mechanistic example alone.

**Note:** Illustrative applications differ in evidence level, and findings from one variant or experimental model do not establish suitability across all disorders associated with the same gene. Across molecular strategies, candidate assessment should consider treatment timing and disease stage, distinguishing prevention of emerging manifestations from improvement or stabilization of established disease. Therapeutic windows may differ across outcomes, as discussed in Section 3.3. Abbreviations: ASO, antisense oligonucleotide; UBE3A-ATS, UBE3A antisense transcript.

## Data Availability

No original experimental or patient-level datasets were generated in this narrative review. The evidence discussed was obtained from the cited scientific literature, publicly available clinical trial registrations, and identified sponsor disclosures.

## Supplementary Materials

The following supporting information can be downloaded at: https://www.mdpi.com/article/10.3390/biology15212010/s1, File S1: Antisense Oligonucleotides in Genetic Epilepsies and Developmental and Epileptic Encephalopathies: Molecular Mechanisms, Translational Evidence and Disease-Modifying Potentia; Table S1: Variant-to-intervention framework for ASO development in genetic epilepsies; Table S2: Translational evidence and principal limitations of ASO programs in genetic epilepsies.

## Abbreviations

AAV	adeno-associated virus
AI	Artificial intelligence
ASMs	antiseizure medications
ASO	antisense oligonucleotide
Bayley-4	Bayley Scales of Infant and Toddler Development, Fourth Edition
CACNA1C	calcium voltage-gated channel subunit alpha1 C
CDKL5	cyclin-dependent kinase-like 5
CLN7	neuronal ceroid lipofuscinosis type 7
CNS	central nervous system
CSF	cerebrospinal fluid
DEE	developmental and epileptic encephalopathy
DNA	deoxyribonucleic acid
EEG	electroencephalography
GNAO1	G protein subunit alpha o1
ILAE	International League Against Epilepsy
KCNQ2	potassium voltage-gated channel subfamily Q member 2
KCNT1	potassium sodium-activated channel subfamily T member 1
MALAT1	metastasis-associated lung adenocarcinoma transcript 1
MDRI	Multidomain Responder Index
MFSD8	major facilitator superfamily domain containing 8
mRNA	messenger RNA
pre-mRNA	precursor messenger RNA
PTBP2	polypyrimidine tract-binding protein 2
RNA	ribonucleic acid
RNase H1	ribonuclease H1
SCN1A	sodium voltage-gated channel alpha subunit 1
SCN2A	sodium voltage-gated channel alpha subunit 2
SCN8A	sodium voltage-gated channel alpha subunit 8
SMN2	survival of motor neuron 2
SOD1	superoxide dismutase 1
STXBP1	syntaxin-binding protein 1
SUDEP	sudden unexpected death in epilepsy
SYNGAP1	synaptic Ras GTPase-activating protein 1
TANGO	targeted augmentation of nuclear gene output
UBE3A	ubiquitin protein ligase E3A
UBE3A-ATS	UBE3A antisense transcript
2′-MOE	2′-O-methoxyethyl

## References

[1] Scheffer I.E., Berkovic S., Capovilla G., Connolly M.B., French J., Guilhoto L., Hirsch E., Jain S., Mathern G.W., Moshé S.L. (2017). ILAE Classification of the Epilepsies: Position Paper of the ILAE Commission for Classification and Terminology. Epilepsia.

[2] Wirrell E.C., Hood V., Knupp K.G., Meskis M.A., Nabbout R., Scheffer I.E., Wilmshurst J., Sullivan J. (2022). International Consensus on Diagnosis and Management of Dravet Syndrome. Epilepsia.

[3] Wolff M., Johannesen K.M., Hedrich U.B.S., Masnada S., Rubboli G., Gardella E., Lesca G., Ville D., Milh M., Villard L. (2017). Genetic and Phenotypic Heterogeneity Suggest Therapeutic Implications in SCN2A-Related Disorders. Brain.

[4] Crooke S.T., Baker B.F., Crooke R.M., Liang X. (2021). Antisense Technology: An Overview and Prospectus. Nat. Rev. Drug Discov..

[5] Lim K.H., Han Z., Jeon H.Y., Kach J., Jing E., Weyn-Vanhentenryck S., Downs M., Corrionero A., Oh R., Scharner J. (2020). Antisense Oligonucleotide Modulation of Non-Productive Alternative Splicing Upregulates Gene Expression. Nat. Commun..

[6] Meng L., Ward A.J., Chun S., Bennett C.F., Beaudet A.L., Rigo F. (2015). Towards a Therapy for Angelman Syndrome by Targeting a Long Non-Coding RNA. Nature.

[7] Finkel R.S., Mercuri E., Darras B.T., Connolly A.M., Kuntz N.L., Kirschner J., Chiriboga C.A., Saito K., Servais L., Tizzano E. (2017). Nusinersen versus Sham Control in Infantile-Onset Spinal Muscular Atrophy. N. Engl. J. Med..

[8] Han Z., Chen C., Christiansen A., Ji S., Lin Q., Anumonwo C., Liu C., Leiser S.C., Meena, Aznarez I. (2020). Antisense Oligonucleotides Increase *Scn1a* Expression and Reduce Seizures and SUDEP Incidence in a Mouse Model of Dravet Syndrome. Sci. Transl. Med..

[9] Laux L., Sullivan J., Perry M.S., Brunklaus A., Desurkar A., Schreiber J.M., Roberts C.M., Knupp K.G., Wheless J.W., Wirrell E.C. (2026). Zorevunersen in Children and Adolescents with Dravet Syndrome. N. Engl. J. Med..

[10] Wagner M., Berecki G., Fazeli W., Nussbaum C., Flemmer A.W., Frizzo S., Heer F., Heinen F., Horton R., Jacotin H. (2025). Antisense Oligonucleotide Treatment in a Preterm Infant with Early-Onset SCN2A Developmental and Epileptic Encephalopathy. Nat. Med..

[11] Mazur C., Powers B., Zasadny K., Sullivan J.M., Dimant H., Kamme F., Hesterman J., Matson J., Oestergaard M., Seaman M. (2019). Brain Pharmacology of Intrathecal Antisense Oligonucleotides Revealed through Multimodal Imaging. JCI Insight.

[12] Zuberi S.M., Wirrell E., Yozawitz E., Wilmshurst J.M., Specchio N., Riney K., Pressler R., Auvin S., Samia P., Hirsch E. (2022). ILAE Classification and Definition of Epilepsy Syndromes with Onset in Neonates and Infants: Position Statement by the ILAE Task Force on Nosology and Definitions. Epilepsia.

[13] Schüle R., Graessner H., Aartsma-Rus A., van Roon-Mom W.M.C., Synofzik M. (2025). Tailored Antisense Oligonucleotides for Ultrarare CNS Diseases: An Experience-Based Best Practice Framework for Individual Patient Evaluation. Mol. Ther. Nucleic Acids.

[14] Shen W., De Hoyos C.L., Migawa M.T., Vickers T.A., Sun H., Low A., Bell T.A., Rahdar M., Mukhopadhyay S., Hart C.E. (2019). Chemical Modification of PS-ASO Therapeutics Reduces Cellular Protein-Binding and Improves the Therapeutic Index. Nat. Biotechnol..

[15] Liang X.-H., Sun H., Nichols J.G., Crooke S.T. (2017). RNase H1-Dependent Antisense Oligonucleotides Are Robustly Active in Directing RNA Cleavage in Both the Cytoplasm and the Nucleus. Mol. Ther..

[16] Li M., Jancovski N., Jafar-Nejad P., Burbano L.E., Rollo B., Richards K., Drew L., Sedo A., Heighway J., Pachernegg S. (2021). Antisense Oligonucleotide Therapy Reduces Seizures and Extends Life Span in an SCN2A Gain-of-Function Epilepsy Model. J. Clin. Investig..

[17] Kim J., Hu C., Moufawad El Achkar C., Black L.E., Douville J., Larson A., Pendergast M.K., Goldkind S.F., Lee E.A., Kuniholm A. (2019). Patient-Customized Oligonucleotide Therapy for a Rare Genetic Disease. N. Engl. J. Med..

[18] Bennett C.F., Krainer A.R., Cleveland D.W. (2019). Antisense Oligonucleotide Therapies for Neurodegenerative Diseases. Annu. Rev. Neurosci..

[19] Wang S., Sun H., Tanowitz M., Liang X., Crooke S.T. (2017). Intra-Endosomal Trafficking Mediated by Lysobisphosphatidic Acid Contributes to Intracellular Release of Phosphorothioate-Modified Antisense Oligonucleotides. Nucleic Acids Res..

[20] Rigo F., Chun S.J., Norris D.A., Hung G., Lee S., Matson J., Fey R.A., Gaus H., Hua Y., Grundy J.S. (2014). Pharmacology of a Central Nervous System Delivered 2′-O-Methoxyethyl–Modified Survival of Motor Neuron Splicing Oligonucleotide in Mice and Nonhuman Primates. J. Pharmacol. Exp. Ther..

[21] Kuijper E.C., van der Graaf L., Pepers B.A., Guimarães Ramos M., Korhorn S., Toonen L.J.A., Cats D., Buijsen R.A.M., Mina E., Mei H. (2025). Determining Off-Target Effects of Splice-Switching Antisense Oligonucleotides Using Short Read RNAseq in Neuronally Differentiated Human Induced Pluripotent Stem Cells. Hum. Mol. Genet..

[22] Lenk G.M., Jafar-Nejad P., Hill S.F., Huffman L.D., Smolen C.E., Wagnon J.L., Petit H., Yu W., Ziobro J., Bhatia K. (2020). Scn8a Antisense Oligonucleotide Is Protective in Mouse Models of SCN8A Encephalopathy and Dravet Syndrome. Ann. Neurol..

[23] Burbano L.E., Li M., Jancovski N., Jafar-Nejad P., Richards K., Sedo A., Soriano A., Rollo B., Jia L., Gazina E.V. (2022). Antisense Oligonucleotide Therapy for KCNT1 Encephalopathy. JCI Insight.

[24] Orhan G., Bock M., Schepers D., Ilina E.I., Reichel S.N., Löffler H., Jezutkovic N., Weckhuysen S., Mandelstam S., Suls A. (2014). Dominant-negative Effects of *KCNQ2* Mutations Are Associated with Epileptic Encephalopathy. Ann. Neurol..

[25] Kim-McManus O., Mignon L., Douville J., Pu H., Parisien C., Glass S., Bennett C.F., Celso J., Robbins K., Ung H. (2026). Individualized Antisense Oligonucleotides for SCN2A-Related Developmental Epileptic Encephalopathy. Nat. Med..

[26] Berecki G., Tao E., Howell K.B., Coorg R.K., Andersen E., Kahlig K., Wolff M., Corry B., Petrou S. (2025). Nav1.2 Channel Mutations Preventing Fast Inactivation Lead to *SCN2A* Encephalopathy. Brain.

[27] Kim J., Woo S., De Gusmao C.M., Zhao B., Chin D.H., DiDonato R.L., Nguyen M.A., Nakayama T., Hu C.A., Soucy A. (2023). A Framework for Individualized Splice-Switching Oligonucleotide Therapy. Nature.

[28] Scheffer I.E., French J., Valente K.D., Auvin S., Cross J.H., Specchio N. (2025). Operational Definition of Developmental and Epileptic Encephalopathies to Underpin the Design of Therapeutic Trials. Epilepsia.

[29] Golinski S.R., Soriano K., Briegel A.C., Burke M.C., Tang S., Carvill G.L., Sherrill E., Lentucci C., Yu T.W., Nakayama T. (2026). RNA Targeting Therapy for a Prenatally Enriched Potassium Channel Associated with Severe Childhood Epilepsy and Premature Death. Nat. Commun..

[30] Hill S.F., Yu W., Ziobro J., Chalasani S., Reger F., Meisler M.H. (2024). Long-Term Downregulation of the Sodium Channel Gene Scn8a Is Therapeutic in Mouse Models of SCN8A Epilepsy. Ann. Neurol..

[31] Silva-Santos S., van Woerden G.M., Bruinsma C.F., Mientjes E., Jolfaei M.A., Distel B., Kushner S.A., Elgersma Y. (2015). Ube3a Reinstatement Identifies Distinct Developmental Windows in a Murine Angelman Syndrome Model. J. Clin. Investig..

[32] Rotaru D.C., van Woerden G.M., Wallaard I., Elgersma Y. (2018). Adult Ube3a Gene Reinstatement Restores the Electrophysiological Deficits of Prefrontal Cortex Layer 5 Neurons in a Mouse Model of Angelman Syndrome. J. Neurosci..

[33] Milazzo C., Mientjes E.J., Wallaard I., Rasmussen S.V., Erichsen K.D., Kakunuri T., Van Der Sman A.S.E., Kremer T., Miller M.T., Hoener M.C. (2021). Antisense Oligonucleotide Treatment Rescues UBE3A Expression and Multiple Phenotypes of an Angelman Syndrome Mouse Model. JCI Insight.

[34] Lee D., Chen W., Kaku H.N., Zhuo X., Chao E.S., Soriano A., Kuncheria A., Flores S., Kim J.H., Rivera A. (2023). Antisense Oligonucleotide Therapy Rescues Disturbed Brain Rhythms and Sleep in Juvenile and Adult Mouse Models of Angelman Syndrome. eLife.

[35] Nakayama T., El Achkar C.M., Burbano L.E., Quraishi I.H., Wu J., Li M., Asami Y., Golinski S.R., Sherrill E., Goodlett B.D. (2026). Antisense Oligonucleotide-Mediated Knockdown Therapy in Two Infants with Severe KCNT1 Epileptic Encephalopathy. Nat. Med..

[36] D’Gama A.M., Mulhern S., Sheidley B.R., Boodhoo F., Buts S., Chandler N.J., Cobb J., Curtis M., Higginbotham E.J., Holland J. (2023). Evaluation of the Feasibility, Diagnostic Yield, and Clinical Utility of Rapid Genome Sequencing in Infantile Epilepsy (Gene-STEPS): An International, Multicentre, Pilot Cohort Study. Lancet Neurol..

[37] Mitchell J.W., Sossi F., Miller I., Jaber P.B., Das-Gupta Z., Fialho L.S., Amos A., Austin J.K., Badzik S., Baker G. (2024). Development of an International Standard Set of Outcomes and Measurement Methods for Routine Practice for Infants, Children, and Adolescents with Epilepsy: The International Consortium for Health Outcomes Measurement Consensus Recommendations. Epilepsia.

[38] Stoke Therapeutics, Inc. EMPEROR: A Multicenter, Randomized, Double-Blind, Sham-Controlled, Parallel Group, Phase 3 Study Evaluating the Efficacy, Safety, and Tolerability of Zorevunersen (STK-001) in Patients with Dravet Syndrome. https://clinicaltrials.gov/study/NCT06872125.

[39] Stoke Therapeutics, Inc. Stoke Therapeutics Announces Completion of Enrollment of 162 Patients into the Phase 3 EMPEROR Study of Zorevunersen, an Investigational Medicine for the Treatment of Dravet Syndrome. https://investor.stoketherapeutics.com/news-releases/news-release-details/stoke-therapeutics-announces-completion-enrollment-162-patients.

[40] Ionis Pharmaceuticals, Inc. Ionis Doses First Participant in Phase 1-2 ASCEND Study of ION337 in Dravet Syndrome. https://ir.ionis.com/news-releases/news-release-details/ionis-doses-first-participant-phase-1-2-ascend-study-ion337.

[41] ClinicalTrials.gov ASCEND: Safety and Tolerability of ION337 for the Treatment of Dravet Syndrome. https://clinicaltrials.gov/study/NCT07531745.

[42] Praxis Precision Medicines, Inc. Praxis Precision Medicines Announces Positive Results from the EMBRAVE Part A Trial of Elsunersen in Patients with SCN2A Early-Onset Developmental and Epileptic Encephalopathy. https://investors.praxismedicines.com/news-releases/news-release-details/praxis-precision-medicines-announces-positive-results-embrave.

[43] Praxis Precision Medicines, Inc. A Multi-Center, Single-Arm Clinical Trial to Investigate the Efficacy and Safety of Elsunersen in Pediatric Participants with Early Onset SCN2A Developmental and Epileptic Encephalopathy (NCT07019922). NCT07019922.

[44] Hill S.F., Jafar-Nejad P., Rigo F., Meisler M.H. (2023). Reduction of Kcnt1 Is Therapeutic in Mouse Models of SCN1A and SCN8A Epilepsy. Front. Neurosci..

[45] ClinicalTrials.gov A First-in-Human Study of S230815 in Pediatric Participants with KCNT1-Related Developmental and Epileptic Encephalopathy (KANDLE; NCT07227857). NCT07227857.

[46] Servier Pharmaceuticals Servier Announces First Patient Enrolled in Phase Ib/II Study for the Treatment of a Rare Pediatric Epilepsy Syndrome in the U.S. https://servier.mediaroom.com/2026-06-25-Servier-Announces-First-Patient-Enrolled-in-Phase-Ib-II-Study-for-the-Treatment-of-a-Rare-Pediatric-Epilepsy-Syndrome-in-the-U-S.

[47] Hipp J.F., Bacino C.A., Bird L.M., Bruenig-Traebert I., Chan D., de Wit M.C., Fontoura P., Hooper G., Jagasia R., Krishnan M.L. (2025). The UBE3A-ATS Antisense Oligonucleotide Rugonersen in Children with Angelman Syndrome: A Phase 1 Trial. Nat. Med..

[48] Ionis Pharmaceuticals, Inc. Ionis Announces Positive Detailed Results from the HALOS Study of ION582 in People with Angelman Syndrome. https://ir.ionis.com/news-releases/news-release-details/ionis-announces-positive-detailed-results-halos-study-ion582.

[49] ClinicalTrials.gov HALOS: A Safety, Tolerability, Pharmacokinetics and Pharmacodynamics Study of Multiple Ascending Doses of ION582 in Participants with Angelman Syndrome. https://clinicaltrials.gov/study/NCT05127226.

[50] ClinicalTrials.gov REVEAL: A Phase 3 Study of Obudanersen (ION582) in Angelman Syndrome. https://clinicaltrials.gov/study/NCT06914609.

[51] Ionis Pharmaceuticals, Inc. Ionis Completes Enrollment in Pivotal Cohort of the Phase 3 REVEAL Study Evaluating Obudanersen in Angelman Syndrome. https://ir.ionis.com/node/34841.

[52] ClinicalTrials.gov CHAMPION: A Study to Evaluate the Efficacy and Safety of Obudanersen (ION582) in Children and Adults with Angelman Syndrome (AS). https://clinicaltrials.gov/study/NCT07782827.

[53] Ionis Pharmaceuticals, Inc. Ionis Receives U.S. FDA Breakthrough Therapy Designation for ION582 in Angelman Syndrome. https://ir.ionis.com/news-releases/news-release-details/ionis-receives-us-fda-breakthrough-therapy-designation-ion582.

[54] Ultragenyx Pharmaceutical Inc. A Phase 3, Randomized, Double-Blind, Sham-Controlled Study Investigating the Efficacy and Safety of GTX-102 in Pediatric Subjects with Angelman Syndrome (NCT06617429). NCT06617429.

[55] Ultragenyx Pharmaceutical Inc Ultragenyx Announces Phase 3 Aspire Results in Angelman Syndrome. https://ir.ultragenyx.com/news-releases/news-release-details/ultragenyx-announces-phase-3-aspire-results-angelman-syndrome.

[56] Keute M., Miller M.T., Krishnan M.L., Sadhwani A., Chamberlain S., Thibert R.L., Tan W.-H., Bird L.M., Hipp J.F. (2021). Angelman Syndrome Genotypes Manifest Varying Degrees of Clinical Severity and Developmental Impairment. Mol. Psychiatry.

[57] Powers S., Anderson K.N., Tan W.-H., Tillmann J., Daniel M., Thurm A., Farmer C., Clinch S., Wheeler A.C., Sadhwani A. (2026). Developing Meaningful Score Differences for the Bayley-4 and Vineland-3 in Angelman Syndrome Using a Delphi Panel. Value Health.

[58] Correale C., Specchio N., Downs J., Cross J.H., Cappelletti S., Palacio-Navarro A., Geuens S., Valente K., Brunklaus A., Reilly C. (2026). Cognitive and Behavioral Clinical Outcome Assessments in Children with Developmental and Epileptic Encephalopathies: Issues and Instruments. Epilepsia.

[59] GeneTx Biotherapeutics LLC, Ultragenyx Pharmaceutical Inc GeneTx and Ultragenyx Announce Positive Interim Phase 1/2 Data on Investigational GTX-102 Demonstrating Improvement in Patients with Angelman Syndrome. https://www.sec.gov/Archives/edgar/data/1515673/000095017020000088/rare-20201026-ex99_1.htm.

[60] Aartsma-Rus A., Garanto A., Van Roon-Mom W., Mcconnell E.M., Suslovitch V., Yan W.X., Watts J.K., Yu T.W. (2023). on behalf of the N = 1 Collaborative. Consensus Guidelines for the Design and In Vitro Preclinical Efficacy Testing N-of-1 Exon Skipping Antisense Oligonucleotides. Nucleic Acid. Ther..

[61] ClinicalTrials.gov An Open-Label, Non-Randomized Study to Evaluate the Efficacy and Safety of ASO-GNAO1 (Tianasen) in Patients with GNAO1-Encephalopathy with Epilepsy and Movement Disorders Following Repeated Intrathecal Dose Escalation. https://clinicaltrials.gov/study/NCT07363603.

[62] Shomer I., Mor N., Raviv S., Budick-Harmelin N., Matchevich T., Avkin-Nachum S., Rais Y., Haffner-Krausz R., Haimovich A., Ziv A. (2025). Personalized Allele-Specific Antisense Oligonucleotides for GNAO1-Neurodevelopmental Disorder. Mol. Ther. Nucleic Acids.

[63] Lovett A., Chary S., Babu S., Bruneteau G., Glass J.D., Karlsborg M., Ladha S., Mayl K., McDermott C., Bucelli R.C. (2025). Serious Neurologic Adverse Events in Tofersen Clinical Trials for Amyotrophic Lateral Sclerosis. Muscle Nerve.

[64] Biogen Inc. SPINRAZA (Nusinersen) Injection, for Intrathecal Use: Full Prescribing Information. https://dailymed.nlm.nih.gov/dailymed/fda/fdaDrugXsl.cfm?setid=dd70cd5f-b0fc-4ba4-a5ea-89a34778bd94&type=display.

[65] Crooke S.T., Baker B.F., Kwoh T.J., Cheng W., Schulz D.J., Xia S., Salgado N., Bui H.-H., Hart C.E., Burel S.A. (2016). Integrated Safety Assessment of 2′-O-Methoxyethyl Chimeric Antisense Oligonucleotides in NonHuman Primates and Healthy Human Volunteers. Mol. Ther..

[66] Haché M., Swoboda K.J., Sethna N., Farrow-Gillespie A., Khandji A., Xia S., Bishop K.M. (2016). Intrathecal Injections in Children with Spinal Muscular Atrophy. J. Child Neurol..

[67] Carson V.J., Young M., Brigatti K.W., Robinson D.L., Reed R.M., Sohn J., Petrillo M., Farwell W., Miller F., Strauss K.A. (2022). Nusinersen by Subcutaneous Intrathecal Catheter for Symptomatic Spinal Muscular Atrophy Patients with Complex Spine Anatomy. Muscle Nerve.

[68] Barker S.J., Thayer M.B., Kim C., Tatarakis D., Simon M.J., Dial R., Nilewski L., Wells R.C., Zhou Y., Afetian M. (2024). Targeting the Transferrin Receptor to Transport Antisense Oligonucleotides across the Mammalian Blood-Brain Barrier. Sci. Transl. Med..

[69] Cheerie D., Meserve M.M., Beijer D., Kaiwar C., Newton L., Taylor Tavares A.L., Verran A.S., Sherrill E., Leonard S., Sanders S.J. (2025). Consensus Guidelines for Assessing Eligibility of Pathogenic DNA Variants for Antisense Oligonucleotide Treatments. Am. J. Hum. Genet..

[70] Chen X., Birey F., Li M.-Y., Revah O., Levy R., Thete M.V., Reis N., Kaganovsky K., Onesto M., Sakai N. (2024). Antisense Oligonucleotide Therapeutic Approach for Timothy Syndrome. Nature.

[71] Dawicki-McKenna J.M., Felix A.J., Waxman E.A., Cheng C., Amado D.A., Ranum P.T., Bogush A., Dungan L.V., Maguire J.A., Gagne A.L. (2023). Mapping PTBP2 Binding in Human Brain Identifies SYNGAP1 as a Target for Therapeutic Splice Switching. Nat. Commun..

[72] Felix A.J., Brown B.L., Marotta N., Gessner M.J., Houserova M., Huerta-Ocampo I., Wilson T., Randell R., Dawicki-McKenna J.M., Reinhardt D. (2026). Translatable Electrophysiological and Behavioral Abnormalities in a Humanized Model of SYNGAP1-Disorder. Mol. Psychiatry.

[73] Belgrad J., McConnell E., Leonard S., Nolen N., Lauffer M.C., Watts J.K., Yu T., Yan W.X., Aartsma-Rus A. (2025). The N=1 Collaborative: Advancing Customized Nucleic Acid Therapies through Collaboration and Data Sharing. Nucleic Acids Res..

[74] Aartsma-Rus A., de Gusmao C.M., Demarest S., Wagner M., Marckmann G., Kogel C., Favresse R., Schuele R., Synofzik M., Graessner H. (2026). Guidance on Communication and Informed Consent with Patients and Their Families for Experimental Individualized Treatments. Am. J. Hum. Genet..

[75] Synofzik M., van Roon-Mom W.M.C., Marckmann G., van Duyvenvoorde H.A., Graessner H., Schüle R., Aartsma-Rus A. (2022). Preparing N-of-1 Antisense Oligonucleotide Treatments for Rare Neurological Diseases in Europe: Genetic, Regulatory, and Ethical Perspectives. Nucleic Acid Ther..

[76] U.S. Food and Drug Administration IND Submissions for Individualized Antisense Oligonucleotide Drug Products: Administrative and Procedural Recommendations. https://www.fda.gov/regulatory-information/search-fda-guidance-documents/ind-submissions-individualized-antisense-oligonucleotide-drug-products-administrative-and-procedural.

