# Supplementary material for: Antisense Oligonucleotidesin Genetic Epilepsies and Developmental and Epileptic Encephalopathies: Molecular Mechanisms, Translational Evidence and Disease-Modifying Potential
Source: Biology (Basel). 2026 Nov 5;15(21):2010. doi: 10.3390/biology15212010 (PMC13762165; doi:10.3390/biology15212010)
Supplement: Supplementary file 1 [file biology-15-02010-s001.zip › Supplementary File S1_Literature Search Strategy, Evidence Identification, and Selection Principles_R2.pdf]

Supplementary File S1.

Antisense Oligonucleotides in Genetic Epilepsies and Developmental and Epileptic Encephalopathies: Molecular Mechanisms, Translational Evidence and Disease-Modifying Potential

Literature Search Strategy, Evidence Identification, and Selection Principles

S1. Scope and design

A focused, non-systematic bibliographic search was used to support a narrative review of antisense oligonucleotides (ASOs) in genetic epilepsies and developmental and epileptic encephalopathies. The supplementary searches were undertaken during manuscript revision to document coverage, identify potentially relevant studies, and verify selected mechanistic and clinical claims. They were not designed to provide exhaustive evidence ascertainment, a PRISMA screening flow, a study-level risk-of-bias assessment, or pooled effect estimates.

The four supplementary PubMed searches were performed on October 30, 2026 during manuscript revision. The original narrative review retained a fixed evidence cut-off of September 28, 2026: scientific and clinical information considered for the fixed review period had to be publicly available by that date. The search execution date and the dates of online access do not extend this cut-off. The supplementary searches retrospectively assess coverage of earlier information and must not be represented as the search conducted before the original manuscript submission.

S2. PubMed supplementary searches

Four complementary searches of PubMed title/abstract fields were carried out on **October 30, 2026**. The expressions below are the author-supplied search strings used for the supplementary search. Each query was searched without restrictions on language, publication type, or full-text access; first-publication dates were considered for the fixed September 28, 2026 evidence cut-off when determining whether retrieved evidence was temporally relevant. Because .nbib exports do not embed the PubMed Search Details translation, no additional PubMed-translated terms are attributed to these searches.

Query	Emphasis	Retrieved records
S1	ASO mechanisms and genetic epilepsies	193
S2	Gene-focused ASO programs	204
S3	Individualized ASO, N-of-1, and milasen	44
S4	CNS delivery, pharmacokinetics, and distribution	564

Total exported records: 1,005. **Repeated PMID occurrences across search sets:** 121. **Unique PMIDs:** 884.

S1. ASO mechanisms and genetic epilepsies

("antisense oligonucleotide*"[tiab] OR "splice-switching"[tiab] OR "RNA-targeted"[tiab] OR "gapmer*"[tiab]) AND ("epilep*"[tiab] OR "developmental and epileptic encephalopath*"[tiab] OR "Dravet syndrome"[tiab] OR "Angelman syndrome"[tiab])

S2. Gene-focused ASO programs

("SCN1A"[tiab] OR "SCN2A"[tiab] OR "SCN8A"[tiab] OR "KCNT1"[tiab] OR "UBE3A"[tiab] OR "MFSD8"[tiab] OR "GNAO1"[tiab] OR "STXBP1"[tiab] OR "CDKL5"[tiab] OR "SYNGAP1"[tiab]) AND ("antisense"[tiab] OR "oligonucleotide*"[tiab] OR "splice modulation"[tiab] OR "RNA-targeted"[tiab])

S3. Individualized ASO, N-of-1, and milasen

(milasen[tiab] OR MFSD8[tiab] OR CLN7[tiab] OR "patient-customized"[tiab] OR "individualized antisense"[tiab] OR "N-of-1"[tiab]) AND (oligonucleotide*[tiab] OR antisense[tiab] OR "splice-switching"[tiab])

S4. CNS delivery, pharmacokinetics, and distribution

("antisense oligonucleotide"[tiab] OR "antisense oligonucleotides"[tiab] OR ASO[tiab]) AND ("blood-brain barrier"[tiab] OR "transferrin receptor"[tiab] OR intrathecal[tiab] OR "endosomal escape"[tiab] OR "central nervous system delivery"[tiab])

Deduplication was performed by PMID across all four exports. For searches S3 and S4, the corresponding PubMed CSV exports contained the same 44 and 564 PMIDs, respectively, as the .nbib exports.

S3. Evidence identification beyond indexed articles

Beyond indexed literature, targeted checks were made of ClinicalTrials.gov using gene names, ASO compound/program names, and trial identifiers (including SCN1A, SCN2A, KCNT1, UBE3A, GNAO1, zorevunersen, elsunersen, valeriasen, obudanersen, ION337, S230815, and tianasen). Official dated sponsor communications were consulted for programs without complete peer-reviewed clinical reports (including announcements from Ionis, Stoke, Praxis, Ultragenyx/GeneTx, and Servier). Registry records were used to document trial design, registration and planned outcomes, not to infer efficacy; sponsor announcements were classified as non-peer-reviewed disclosures. For the historical evidence cut-off, trial information first added or revised after **September 28, 2026** must not be treated as though it was available before that date. This targeted program verification was not an exhaustive search of every trial registry or sponsor website.

S4. Narrative evidence selection principles

Primary relevance: Studies directly addressing sequence-directed RNA modulation in a genetic epilepsy or developmental and epileptic encephalopathy, including functional-genetic characterization and relevant pharmacodynamic outcomes.

Translational context: Relevant studies in other neurological conditions where they inform ASO chemistry, central delivery, intracellular availability, tolerability, monitoring, or individualized development.

Evidence hierarchy: Distinction between preclinical studies, individual prospective treatments, uncontrolled clinical observations, controlled trial reports, trial registrations, and sponsor disclosures.

Interpretation: Emphasis on compound-specific exposure, functional rescue, seizure outcomes, development/adaptive function, safety, and the distinction between target engagement and disease modification.

Selection was purposive rather than exhaustive. Retrieved search records should not be interpreted as the number of papers screened, included, or excluded through a systematic review process. No formal risk-of-bias scoring, independent duplicate screening, systematic quality grading, or meta-analysis was conducted. The selection may therefore be affected by publication and selection bias.

S5. Evidence cut-off check

Of the 884 unique PubMed records, 11 had an October 2026 electronic publication date in the DEP field of the supplied .nbib exports, later than the evidence cut-off. They were flagged as post-cut-off records and were not used to extend the fixed evidence period. A further chapter was flagged separately on the basis of a publisher-verified September 30, 2026 eBook date (below). These are date checks on retrieved records, not a count of articles excluded after systematic eligibility screening. The remaining records cannot automatically be assumed eligible, scientifically pertinent, or included. The earliest verifiable public availability date was preferred over a later print-issue date.

PMID	First e-publication	Title
42939560	2026-10-02	SCN1A-Related Epilepsies: Clinical Spectrum and Mechanisms
42829049	2026-10-03	Delivery architectures for oligonucleotide therapeutics in genetic and rare diseases: GalNAc-based hepatocyte targeting and extrahepatic translation
42842141	2026-10-07	Advances in Extracellular Vesicle-Based Innovative Drugs Targeting Alzheimer's Disease
42865191	2026-10-07	Neurovascular Modulation of the Blood-brain Barrier Using Focused Ultrasound for Antisense Therapy Delivery in Amyotrophic Lateral Sclerosis
42850886	2026-10-08	Sustained relief of chronic pain via a Na(v)1.7-targeting ASO-siRNA conjugate in rats
42941401	2026-10-13	Precision Therapeutics for Monogenic Epilepsies: From Molecular Mechanisms to Disease Modification in Developmental and Epileptic Encephalopathies and Neurodevelopmental Disorders with Epilepsy
42874942	2026-10-14	Zein-based hybrid nanocarriers for non-viral gene delivery: A review
42938424	2026-10-20	Genetic Convergence of Autism Spectrum Disorder and Epilepsy: From Molecular Mechanisms to Precision Therapeutics
42914159	2026-10-22	Challenges in Gene-Directed Therapies for Rare Neurodevelopmental Disorders Using HNRNPU as an Exemplar
42910517	2026-10-22	Characterizing NaV1.7 antisense analgesia through antagonist binding, mRNA expression, and neuraxial

PMID	First e-publication	Title
		distribution
42911828	2026-10-22	siRNA therapeutics: from hepatic delivery to extrahepatic targeting

One further record (PMID 42814303; Nakamura et al., "Development of Antisense Oligonucleotide Gapmers for Amyotrophic Lateral Sclerosis") lacks a day-level electronic publication date in its PubMed export. Springer lists publication of its containing eBook, Gapmers: Methods and Protocols (DOI: 10.1007/978-1-0716-5476-7), on September 30, 2026. This chapter was therefore flagged separately as post-cut-off on the basis of publisher metadata, in addition to the 11 records dated directly in PubMed.

Publisher verification: https://link.springer.com/book/10.1007/978-1-0716-5476-7 (accessed October 30, 2026). The four epilepsy/neurodevelopment-related October citations identified during analysis of the initial S1/S2 searches are already among the eleven dated records above; they are not an additional group.

S6. Methodological interpretation and limitations

These counts describe record identification, not screening or inclusion. No PRISMA-style flow diagram, duplicate independent screening, prespecified systematic-review protocol, formal risk-of-bias assessment, SANRA scoring, or meta-analysis was conducted. PubMed alone cannot capture sponsor-reported results or all program registrations; targeted searching may miss relevant publications. The resulting evidence selection is purposive and may be subject to selection or publication bias.

The original narrative evidence appraisal and the supplementary searches performed after manuscript submission are distinct. October retrievals may identify publications first available before the cut-off, but do not retroactively establish that a systematic search was undertaken in September. For therapeutic programs, the date of first posting and historically available registry information should be considered independently of later registration updates. Any newly identified studies first made public after September 28, 2026 were not incorporated into this fixed-cut-off evidence set.
