# Supplementary material for: Antisense Oligonucleotidesin Genetic Epilepsies and Developmental and Epileptic Encephalopathies: Molecular Mechanisms, Translational Evidence and Disease-Modifying Potential
Source: Biology (Basel). 2026 Nov 5;15(21):2010. doi: 10.3390/biology15212010 (PMC13762165; doi:10.3390/biology15212010)
Supplement: Supplementary file 1 [file biology-15-02010-s001.zip › Table S1_R1.pdf]

Table S1. Variant-to-intervention framework for ASO development in genetic epilepsies.

Molecular situation	Intended direction of intervention	Illustrative application	Principal validation requirement
Haploinsufficiency with a targetable non-productive splice event	Increase productive transcript and functional protein	*SCN1A* augmentation [5,8]	Demonstrate useful protein output and assess the consequences of increasing expression from each available allele.
Gain of function with a tolerable reduction window	Partially lower the protein-coding transcript	Selected *SCN2A, SCN8A* and *KCNT1* models [16,22,23]	Establish functional rescue across doses while preserving sufficient physiological activity.
Pathogenic allele distinguishable by an informative sequence	Preferentially lower the pathogenic transcript	Individualized *SCN2A* ASOs [25]	Establish haplotype phase and measure suppression of both alleles separately.
Functional allele subject to regulatory silencing	Target the repressive antisense transcript	*UBE3A-ATS* lowering in Angelman syndrome [6]	Confirm a reactivatable allele and appropriate restoration of expression.
Pathogenic abnormal splicing	Redirect processing towards a useful transcript	*MFSD8* splice correction with milasen [17]	Demonstrate correction of the RNA defect and downstream functional rescue.
Dominant-negative effect or unresolved functional mechanism	Compare mechanism-specific strategies before selecting augmentation or suppression	*KCNQ2* as an illustration of dominant-negative channel dysfunction [24]	Establish how changing mutant and wild-type expression affects net function; an ASO strategy is not established by the mechanistic example alone.

Note: Illustrative applications differ in evidence level, and findings from one variant or experimental model do not establish suitability across all disorders associated with the same gene. Across molecular strategies, candidate assessment should consider treatment timing and disease stage, distinguishing prevention of emerging manifestations from improvement or stabilization of established disease. Therapeutic windows may differ across outcomes, as discussed in Section 3.3. **Abbreviations:** ASO, antisense oligonucleotide; UBE3A-ATS, UBE3A antisense transcript.
