# Supplementary material for: Antisense Oligonucleotidesin Genetic Epilepsies and Developmental and Epileptic Encephalopathies: Molecular Mechanisms, Translational Evidence and Disease-Modifying Potential
Source: Biology (Basel). 2026 Nov 5;15(21):2010. doi: 10.3390/biology15212010 (PMC13762165; doi:10.3390/biology15212010)
Supplement: Supplementary file 1 [file biology-15-02010-s001.zip › Table S2_R2.pdf]

Table S2. Translational evidence and principal limitations of ASO programs in genetic epilepsies.

Target or program	Evidence category / reporting status	Principal evidence	Supported inference	Reported safety findings	Principal limitation
SCN1A / zorevunersen	Published preclinical studies; published uncontrolled phase 1/2 studies; registered controlled phase 3 (no outcomes)	Mouse rescue studies [8]; published phase 1/2a studies and open-label extensions [9]; registered sham-controlled phase 3 EMPEROR study [38], with sponsor-reported enrollment completion [39].	Preclinical efficacy and preliminary human clinical signals support controlled evaluation.	Post-lumbar puncture syndrome (25%, initial studies); elevated CSF protein (45%, extensions). Serious events and deaths were reported; attribution requires separate assessment.	Developmental efficacy and human SUDEP reduction remain unconfirmed.
SCN1A / ION337 (ASCEND)	Registered open-label early-phase trial; no efficacy findings	Registered open-label phase 1/2 dose-escalation study; first participant dosed in 2026 [40,41].	Clinical investigation of another splice-modulating *SCN1A* ASO.	No clinical safety outcomes publicly reported at the cutoff.	No controlled efficacy or long-term safety evidence; distinct from zorevunersen.
SCN2A / elsunersen (individual treatment)	Published single-patient treatment	Published treatment of one preterm infant over 20 months [10].	Feasibility and a possible antiseizure signal during combined treatment.	No serious adverse events reported during the published single-patient treatment involving 19 intrathecal administrations.	Uncontrolled observation with concomitant sodium-channel blockers; severe developmental impairment persisted.
SCN2A / elsunersen (EMBRAVE Part A)	Randomized controlled phase 1/2; preliminary sponsor disclosure	Sponsor-reported randomized, sham-controlled phase 1/2 findings: nine children, 24-week controlled period; disclosure dated April 6, 2026 [42].	The reported sham-adjusted seizure reduction supports further evaluation.	No drug-related serious adverse events or discontinuations disclosed by the sponsor; full safety analysis unavailable.	Small sample and sponsor-reported topline results; complete methods and efficacy and safety analyses are needed for full appraisal.
SCN2A / individualized allele-selective ASOs	Published prospective modified-crossover N-of-1 studies (two patients)	Published prospective modified-crossover N-of-1 studies in two boys using distinct allele-selective ASOs [25].	Feasibility of individualized allele-selective treatment, with heterogeneous antiseizure signals.	No ASO-related serious adverse events reported in the two published individualized studies.	No controlled efficacy estimate; different variants, constructs and concurrent treatment changes limit comparison and attribution.
SCN8A lowering	Published preclinical evidence only	Preventive and post-onset ASO treatment in mouse models [22,30].	Preclinical antiseizure and survival benefits support further translational investigation.	Human safety not established; preclinical evidence only.	These experiments do not establish the human benefit-risk balance or the optimal degree of *SCN8A* reduction.
KCNT1 / valeriasen	Published preclinical studies and two-patient clinical report	Mouse studies using experimental *Kcnt1*-targeting ASOs [23]; published valeriasen treatment in two patients [35].	Preclinical efficacy and clinical antiseizure signals are accompanied by an important safety concern.	Ventricular enlargement or hydrocephalus occurred in both treated patients, requiring neurosurgical intervention.	Ventricular enlargement or hydrocephalus was reported in both treated patients; uncontrolled observations do not establish net clinical benefit.
KCNT1 / S230815 (KANDLE)	Registered open-label early-phase trial; no efficacy findings	Registered first-in-human, open-label phase Ib/II KANDLE study [45,46].	Clinical evaluation of a distinct intrathecal *KCNT1*-targeting ASO.	No clinical safety outcomes publicly reported at the cutoff.	No established efficacy or long-term safety data; results cannot be inferred from valeriasen.
UBE3A-ATS / rugonersen	Published uncontrolled clinical study	Published open-label phase 1 TANGELO study involving 61 children [47].	Exploratory EEG and developmental findings support controlled evaluation.	Pyrexia and vomiting reported; serious adverse events occurred, including treatment-related events.	No randomized comparator; natural-history comparisons are vulnerable to confounding, and EEG delta-power changes are not a validated surrogate for clinical benefit.

Target or program	Evidence category / reporting status	Principal evidence	Supported inference	Reported safety findings	Principal limitation
UBE3A-ATS / obudanersen (ION582)	Sponsor-reported uncontrolled findings; registered phase 3 trials without reported efficacy outcomes	HALOS phase 1/2a sponsor-reported findings [48,49]; randomized phase 3 REVEAL study [50,51]; registered single-arm phase 3 CHAMPION study [52].	Sponsor-reported exploratory functional signals support further evaluation; controlled efficacy results were not available at the evidence cutoff.	Favorable preliminary tolerability reported by the sponsor for HALOS; complete published safety data unavailable.	HALOS uncontrolled and sponsor-reported; pivotal efficacy and long-term safety not established.
UBE3A-ATS / apazunersen (Aspire)	Controlled phase 3 trial; sponsor-reported negative topline findings	Registered randomized, double-blind, sham-controlled phase 3 Aspire trial [54]; negative topline findings disclosed on September 2, 2026 [55].	The trial did not demonstrate benefit on the prespecified primary cognitive or key secondary multidomain responder endpoint for the tested regimen and population.	Lower-extremity weakness reported in five early phase 1/2 participants (2020) [59]; Aspire safety described by the sponsor as consistent with earlier experience, without detailed event rates [55].	Sponsor-reported topline findings; restriction to children aged 4–17 years with full maternal deletions limits generalizability. Uncertainties regarding developmental timing, modified dosing, and endpoint sensitivity do not negate the negative primary and key secondary results.
MFSD8 / milasen	Published single-patient treatment	Individualized splice correction and single-patient treatment [17].	Feasibility of variant-specific ASO development and an observed antiseizure signal.	No serious adverse events reported in the published single-patient treatment.	Uncontrolled observation does not establish arrest of neurodegeneration or generalizable efficacy.
GNAO1 / tianasen (ASO-GNAO1)	Registered early-phase clinical study; no efficacy findings	Registered open-label phase 1/2, c.607G>A variant-defined study [61].	Early clinical evaluation of allele-selective *GNAO1* RNA lowering.	No clinical safety outcomes publicly reported at the cutoff.	Very small selected population; no controlled efficacy estimate or established long-term safety.

Evidence categories are not directly interchangeable: preclinical experiments, individual treatments, uncontrolled studies, controlled trials and sponsor disclosures support different levels of inference. Trial registration documents study design, not efficacy. Murine ASOs and clinical compounds listed under the same gene target are not necessarily identical. Milasen is included as a translational precedent in a genetic neurodegenerative disorder with epilepsy. Safety findings are reported according to the original source and the September 28, 2026, evidence cutoff. Absence of reported serious adverse events does not establish long-term safety, particularly in small or uncontrolled studies. Sponsor-reported findings are not equivalent to complete peer-reviewed analyses. No reported safety outcomes indicate that clinical results were unavailable rather than an absence of adverse events. Abbreviations: ASO, antisense oligonucleotide; CSF, cerebrospinal fluid; EEG, electroencephalography; SUDEP, sudden unexpected death in epilepsy; UBE3A-ATS, UBE3A antisense transcript.
